# HDAC3 genetic and pharmacologic inhibition radiosensitizes fusion positive rhabdomyosarcoma by promoting DNA double-strand breaks

**DOI:** 10.1038/s41420-024-02115-y

**Published:** 2024-08-06

**Authors:** Matteo Cassandri, Antonella Porrazzo, Silvia Pomella, Beatrice Noce, Clemens Zwergel, Francesca Antonella Aiello, Francesca Vulcano, Luisa Milazzo, Simona Camero, Deborah Pajalunga, Massimo Spada, Valeria Manzi, Giovanni Luca Gravina, Silvia Codenotti, Michela Piccione, Miriam Tomaciello, Michele Signore, Giovanni Barillari, Cinzia Marchese, Alessandro Fanzani, Biagio De Angelis, Concetta Quintarelli, Christopher R. Vakoc, Eleanor Y. Chen, Francesca Megiorni, Franco Locatelli, Sergio Valente, Antonello Mai, Rossella Rota, Francesco Marampon

**Affiliations:** 1 Department of Radiotherapy, Policlinico Umberto I, “Sapienza” University of Rome, Rome, Italy; 2 Department of Hematology/Oncology, Cell and Gene Therapy, Bambino Gesù Children’s Hospital, IRCCS, Rome, Italy; 3 Department of Clinical Sciences and Translational Medicine, University of Rome Tor Vergata, Rome, Italy; 4 Department of Drug Chemistry and Technologies, “Sapienza” University of Rome, Rome, Italy; 5 Department of Oncology and Molecular Medicine, Istituto Superiore di Sanità, Rome, Italy; 6 Department of Life Sciences, Health and Health Professions, Link Campus University, Rome, Italy; 7 Department of Experimental Medicine, “Sapienza” University of Rome, Rome, Italy; 8 Center of Animal Research and Welfare, Istituto Superiore di Sanità, Rome, Italy; 9 Department of Biotechnological and Applied Clinical Sciences, University of L’Aquila, L’Aquila, Italy; 10 Department of Molecular and Translational Medicine, University of Brescia, Brescia, Italy; 11 Confocal Microscopy Core Facility, Ospedale Pediatrico Bambino Gesù, IRCCS, Rome, Italy; 12 RPPA Unit, Proteomics Area, Core Facilities, Istituto Superiore di Sanità, Rome, Italy; 13 Cold Spring Harbor Laboratory, Cold Spring Harbor, NY USA; 14 Department of Laboratory Medicine and Pathology, University of Washington, Seattle, WA USA; 15 Department of Life Sciences and Public Health, Catholic University of the Sacred Heart, Rome, Italy; 16 Pasteur Institute, Cenci-Bolognetti Foundation, “Sapienza” University of Rome, Rome, Italy

**Keywords:** Sarcoma, Paediatric cancer, Double-strand DNA breaks

## Abstract

Radiotherapy (RT) plays a critical role in the management of rhabdomyosarcoma (RMS), the prevalent soft tissue sarcoma in childhood. The high risk PAX3-FOXO1 fusion-positive subtype (FP-RMS) is often resistant to RT. We have recently demonstrated that inhibition of class-I histone deacetylases (HDACs) radiosensitizes FP-RMS both in vitro and in vivo. However, HDAC inhibitors exhibited limited success on solid tumors in human clinical trials, at least in part due to the presence of off-target effects. Hence, identifying specific HDAC isoforms that can be targeted to radiosensitize FP-RMS is imperative. We, here, found that only HDAC3 silencing, among all class-I HDACs screened by siRNA, radiosensitizes FP-RMS cells by inhibiting colony formation. Thus, we dissected the effects of HDAC3 depletion using CRISPR/Cas9-dependent HDAC3 knock-out (KO) in FP-RMS cells, which resulted in Endoplasmatic Reticulum Stress activation, ERK inactivation, PARP1- and caspase-dependent apoptosis and reduced stemness when combined with irradiation compared to single treatments. HDAC3 loss-of-function increased DNA damage in irradiated cells augmenting H2AX phosphorylation and DNA double-strand breaks (DSBs) and counteracting irradiation-dependent activation of ATM and DNA-Pkcs as well as Rad51 protein induction. Moreover, HDAC3 depletion hampers FP-RMS tumor growth in vivo and maximally inhibits the growth of irradiated tumors compared to single approaches. We, then, developed a new HDAC3 inhibitor, MC4448, which showed specific cell anti-tumor effects and mirrors the radiosensitizing effects of HDAC3 depletion in vitro synergizing with ERKs inhibition. Overall, our findings dissect the pro-survival role of HDAC3 in FP-RMS and suggest HDAC3 genetic or pharmacologic inhibition as a new promising strategy to overcome radioresistance in this tumor.

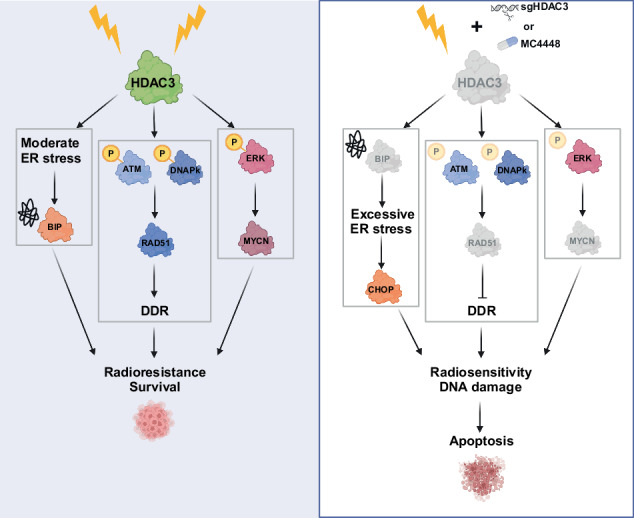

## Introduction

Rhabdomyosarcoma (RMS), a mesenchymal malignancy associated with the skeletal muscle lineage, represents the most common soft-tissue sarcoma in childhood, and accounts for nearly 8% of pediatric solid tumors [1]. The two main histological subtypes of RMS are alveolar (ARMS) and embryonal (ERMS). The majority of ARMS is characterized by the expression of the chimeric fusion oncoprotein PAX3-FOXO1 (P3F) (fusion-positive (FP)-RMS). Fusion-negative (FN)-RMS includes ERMS, the most frequent RMS subtype, and fusion-negative ARMS, both subtypes characterized by a wide range of genetic alterations including mutations of the Receptor-Tyrosine-Kinase- RAS pathway components in more than 50% of cases [1–5]. The overall survival rate for FP-RMS and FN-RMS with metastatic disease remains low, at less than 30% [6]. Adjuvant radiotherapy (RT) is typically administered to RMS patients after surgery (SX) to improve local control [7–10]. However, RMS is intrinsically radioresistant with FP-RMS being the most radioresistant one [9, 10]. Indeed, even the use of hypo-fractionated schedule, with a larger dose per single fraction instead of conventional fractionated fractionation [11], RT fails to overcome the resistance of RMS [12, 13]. Thus, despite recent advancements, approximately one third of individuals with localized RMS experiences local recurrence after RT [14, 15]. Therefore, the elucidation of the radioresistance-related molecular mechanisms is of pivotal importance in establishing novel, more effective, and tolerable combined therapeutic approaches. The HDACs, a group of 18 epigenetic enzymes organized into four classes, have been shown to promote tumor stemness, onset, progression, and resistance to therapies including RT [16]. In line, we have recently shown that the pan-HDACi Belinostat, and the class I-HDACi Entinostat and Romidepsin, all FDA-approved HDACi, radiosensitize FN-RMS and FP-RMS cells, both in vitro and in vivo [17–19]. However, selective HDACs isoform targeting would be ideal to achieve the best therapeutic results with the least toxicity [20]. Recently, HDAC3 has been implicated in the decrease of PAX3-FOXO1 expression, known to be involved in chemo- and radioresistance [21], in FP-RMS models after treatment with Entinostat [22]. However, an involvement of HDAC3 in the response to irradiation in FP-RMS is still to be clarified. In this study, we performed a class-I HDACs screening by genetic silencing followed by irradiation (IR) showing that only HDAC3 depletion enhanced the response to IR in FP-RMS cells. CRISPR/Cas9-mediated HDAC3 knock out (KO) revealed that HDAC3 depletion radiosensitizes FP-RMS cells in vitro. Indeed, combination of HDAC3 depletion with IR hampers cell clonogenic ability, promotes pro-apoptotic ER stress activation, caspase- and PARP-dependent apoptosis, increases DNA damage, and inhibits DNA repair pathway activation compared to each single treatment in FP-RMS cells. Finally, HDAC3 depletion per se impairs tumor growth and amplifies the growth inhibitory effects of IR in vivo. Treatment with our newly developed HDAC3-selective inhibitor MC4448 mirrors the effects of HDAC3 depletion in vitro on irradiated-FP-RMS cells. Altogether, our results highlight the importance of HDAC isoform-specific depletion, suggest a role in DNA damage and repair for HDAC3 in FP-RMS, and support selective inhibition of HDAC3 as a potential adjuvant approach to promote response to RT in this RMS subtype.

## Results

### HDAC3 is overexpressed in RMS and promotes FP-RMS cell survival following exposure to IR

**Fig. 1:**
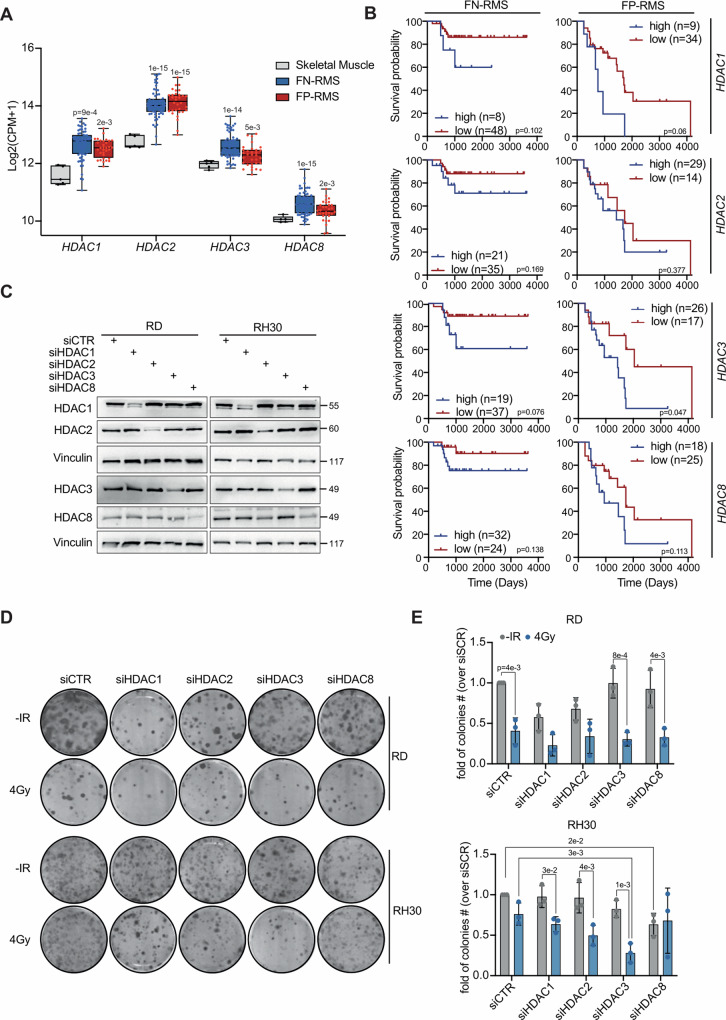
High HDAC3 expression sustain radioresistance in FP-RMS cells. **A** Box plot depicting Class-I HDACs among normal skeletal muscle tissue (*n* = 5), FP- (*n* = 32) and FN-RMS (*n* = 66) patients using GSE108022 dataset. **B** Kaplan–Meyer plot depicting correlation between Class-I HDACs expression level [Williamson dataset and FN- (*n* = 57) and FP-and FN-RMS (*n* = 45) patients’ survival (E-TABM-1202)]. **C** Representative western blot (*n* = 3) of all Class-I HDACs in RD and RH30 cells transfected with either Control (siCTR) or Class-I HDACs siRNAs (siHDAC1, siHDAC2, siHDAC3, siHDAC8) at 48 h post-transfection. Vinculin was used as loading control. **D** Representative images of RD and RH30 colonies stained with crystal violet 12 days post seeding. RMS cells were transfected as in B and 48 h later irradiated with 4 Gy. The cells were processed 6 h post IR. **E** Histograms depicting the plating efficiency of RD and RH30 cells treated as in (**D**). Graph represents the mean of three independent experiments ±SD, one-way ANOVA. Exact *p* values are reported in the histograms.

In recent studies we and others have demonstrated that class-I HDACi are able to radiosensitize RMS cells [18, 19, 22, 23]. Here, we sought to identify specific class-I HDACs responsible for radioresistance in RMS. Firstly, we analyzed the expression levels of class-I HDACs, i.e., HDAC1-3 and 8, in a dataset of primary RMS and normal skeletal muscle samples (GSE108022, [24]) finding a significant upregulation of all members in both RMS subtypes compared to normal muscle tissue (Fig. 1A).

Accordingly, transcriptomic and proteomic data from RMS patient-derived xenografts (PDX) (https://pecan.stjude.cloud/proteinpaint/study/RHB2018) [25] confirmed transcript and protein upregulation of all class-I HDACs vs normal human myoblasts (Supplementary Fig. 1A). Analyzing the impact of class-I HDACs expression on patients’ survival (Williamson dataset, E-TABM-1202, FN-RMS *n* = 56, FP-RMS *n* = 43 [26]) we found no correlation for any HDAC in both RMS subtypes with the exception of *HDAC3*, whose expression significantly correlated with poor prognosis in FP-RMS (Fig. 1B). Then, we transfected two high-risk patient-derived RMS cell lines [27], RD (recurrent FN-RMS) and RH30 (metastatic FP-RMS), with two individual siRNAs targeting each class-I HDACs isoform, both of which reduced HDAC-specific expression compared to cells transfected with scrambled siRNA as control (siCTR) (Supplementary Fig. 1B). The siRNAs with most effective gene knockdown 48 h post transfection were used for subsequent screens in the presence or absence of irradiation (Fig. 1C). We, then, irradiated RMS cells with a sublethal radiation dose (4 Gy) in order to greater highlight radiosensitization effects of HDACs depletion. To quantify cell survival in response to IR, we performed clonogenic assay, as a surrogate survival assay in vitro [28]. In the absence of IR, silencing of any HDAC did not significantly affect colony formation compared to siCTR in both RD and RH30 cells (Fig. 1D, E). The clonogenic ability markedly decreased in RD siCTR with IR vs siCTR cells while IR using 4 Gy of radiation dose, failed to significantly reduce the colony forming efficiency of RH30 siCTR compared to non-irradiated siCTR cells, in line with the lower radiosensitivity of FP-RMS [9, 10] (Fig. 1D, E). Moreover, in RD cells HDAC3 and HDAC8 siRNAs significantly decreased the number of colonies after IR compared to siRNAs alone but, however, no HDAC-specific silencing was able to impact the clonogenic ability after IR with respect to siCTR-irradiated cells (Fig. 1D, E), suggesting that the depletion of any HDAC does not enhance the effects of IR. In RH30 cells, even if colony formation was significantly decreased by the combination of HDAC1, HDAC2, and HDAC3 silencing with IR compared to siRNAs alone, only HDAC3 silencing significantly decreased colony formation compared to both HDAC3 siRNA and IR single treatments (Fig. 1D, E).

Moreover, the effects of CRISPR/Cas9 knockout (KO) of class-I HDACs on the survival of RMS cell lines in DepMap portal (Achilles project, https://depmap.org/portal/achilles) showed that both FP-RMS and FN-RMS cells are strongly dependent from *HDAC3* expression compared to all other class-I HDACs (<0.5 Chronos score) (Supplementary Fig. 1C). Furthermore, RMS cell lines are among the most dependent on *HDAC3* expression among all the tumor cell lines present in the DepMap portal, confirming the importance of HDAC3 in RMS cells survival [29] (Supplementary Fig. 1D). We then take advantage of RNA-seq data in DepMap portal to analyze the expression of HDAC3 in RMS cell lines showing that it is overexpressed in both RMS subtypes compared to normal human fibroblasts (Supplementary Fig. 1E). Moreover, also HDAC3 protein levels appeared upregulated in a panel of RMS cell lines compared to Human Skeletal Muscle Myoblasts (HSMM) (Supplementary Fig. 1F).

Altogether, these data suggest that HDAC3 expression is upregulated in RMS patients’ samples and cell lines, and it has a prognostic role in FP-RMS where it could be involved in resistance to IR.

### CRISPR-mediated HDAC3 KO radiosensitizes FP-RMS cells by inducing Caspase 3/7- and PARP1-dependent apoptosis

**Fig. 2:**
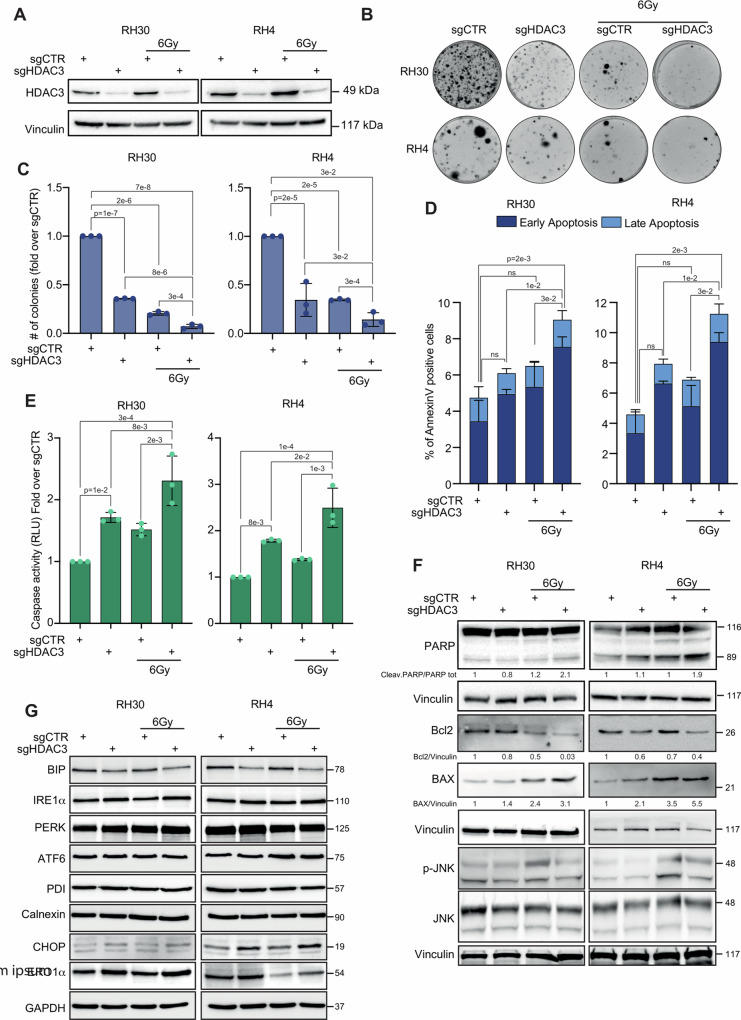
HDAC3 depletion radiosensitizes FP-RMS cells inducing Caspase3/7 and PARP dependent apoptosis. **A** Representative western blot (*n* = 3) depicting HDAC3 protein level of RH30 and RH4 cells stably expressing Cas9 infected with either sgControl (sgCTR) or sgRNA against HDAC3 (sgHDAC3) at 72 h post-infection and then treated or not with 6 Gy of IR. Vinculin was used as loading control. **B** Representative images of RH30 and RH4 colonies stained with crystal violet 12 days post seeding. FP-RMS cells were infected as in (**A**) and 72 h later irradiated or not with 6 Gy. The cells were processed 6 h post IR. **C** Histograms depicting the plating efficiency of RH30 and RH4 treated as in (**B**). Graph represents the mean of three independent experiments ±SD, one-way ANOVA. Exact *p* values are reported in the figure. **D** Histogram depicting the percentage of Annexin-V positive/7-AAD single- and double-positive RH30 and RH4 cells treated as in (**B**). Graphs represent the mean of three independent experiments ±SD, one-way ANOVA. Exact *p* values are reported in the figure. **E** Histogram depicts the quantification of Caspase 3/7 activity RH30 and RH4 cells treated as in (**B**). Graphs represent the mean of three independent experiments ±SD, one-way ANOVA. Exact *p* values are reported in the figure. **F** Representative western blot (*n* = 3) depicting levels of Cleaved- PARP1, Bcl2; BAX, p-JNK, and JNK of RH30 and RH4 cells infected with either sgCTR or sgHDAC3 at 72 h post-infection and the treated or not with 6 Gy of IR. Vinculin was used as loading control. **G** Representative western blot (*n* = 3) depicting levels of BIP, IRE1α, PERK, ATF6, PDI, Calnexin, CHOP and ERO1α of RH30 and RH4 cells infected with either sgCTR or sgHDAC3 at 72 h post-infection and the treated or not with 6 Gy of IR. GAPDH was used as loading control.

To confirm the involvement of HDAC3 in the resistance to IR in FP-RMS we performed functional and molecular studies after *HDAC3* KO using CRISPR/Cas9 mediated gene editing. To this end, RH30 and RH4 FP-RMS cell lines, engineered to stably expressing Cas9, were transduced either with a lentiviral vector expressing dual sgRNAs targeting HDAC3 (sgHDAC3), to increase KO efficiency, or an empty-vector as control (sgCTR), as described in our previous work [29]. The efficiency of HDAC3 depletion 72 h post-infection for the bulk polyclonal cell population for the two cell lines is shown in Fig. 2A.

We then irradiated the HDAC3 KO cells with 6 Gy of IR, which corresponding to the standard-of-care RT dosage clinically [12] and performed colony formation assay.

In contrast to the results obtained with siRNAs, CRISPR/Cas9-mediated HDAC3 depletion markedly reduced cell clonogenic efficiency compared to non-irradiated controls in both cell lines. Similarly, 6 Gy of IR significantly affected colony formation *vs* non-irradiated sgCTR. Noteworthy, combining HDAC3 KO with IR amplified the inhibition of colony formation compared to either HDAC3 depletion or IR alone (Fig. 2B, C).

To functionally assess whether the strong reduction in cell survival due to HDAC3 KO and IR combination was caused by apoptotic cell death, we performed Annexin V staining in RH30 and RH4 cells 24 h after 6 Gy IR. We found that neither HDAC3 depletion nor IR impacted significantly on the Annexin V cells positivity when applied alone on both cell lines compared to non-irradiated sgCTR cells (for RH30 *p* = 0.18 and *p* = 0.1; *p* = 0.19 and *p* = 0.1 for RH4 respectively) (Fig. 2D). Conversely, the combination of HDAC3 loss-of-function and IR efficiently enhanced apoptotic cell death in RH30 and RH4 cells particularly increasing the early apoptotic cell population respect to the late apoptotic one (Fig. 2D).

Accordingly, sgHDAC3 cells showed additive effects with IR on Caspase 3/7 activity and PARP1 cleavage compared with single treatments in both cell lines while no significant modulation was seen in sgHDAC3 and irradiated cells alone *vs* non-irradiated control cells (Fig. 2E, F). In line, levels of the anti-apoptotic protein Bcl2 were further downregulated while those of the pro-apoptotic protein BAX upregulated in irradiated cells depleted for HDAC3 compared to single treatments (Fig. 2F). Moreover, HDAC3 KO counteracted the IR-induced JNKs phosphorylation/activation (Fig. 2F), which inhibition has been recently shown to enhance radiosensitivity and IR-induced apoptosis [30, 31].

The activation of endoplasmic reticulum (ER) stress, trough the unfolded protein response (UPR) cascade, is known to potentially sustain IR-induced apoptosis [32]. As illustrated in Fig. 2G, sgHDAC3 cells downregulated basal expression levels of BIP, primary sensor and inhibitor of UPR cascade, and upregulated the expression of pro-apoptotic UPR downstream C/EBP Homologous Protein (CHOP) transcription factor and of its downstream target Endoplasmic Reticulum Oxidoreductase 1 Alpha (ERO1α) (Fig. 2G). No changes occurred in the expression of total levels of RNA-like endoplasmic reticulum kinase (PERK), activating transcription factor 6α (ATF6), and inositol-requiring enzyme-1α (IRE1α), the three main mediators of UPR, as well as of pro-survival effectors calnexin [33, 34] and Protein Disulfide Isomerase (PDI) [35, 36] (Fig. 2G).

These data suggest that the apoptotic phenotype of irradiated HDAC3 KO RH30 and RH4 cells could be mediated, at least in part, by both Caspase 3/7 and PARP pathways, suggesting a role for the activation of pro-apoptotic signals partly by ER stress response.

Moreover, IR alone induced G2/M accumulation, as we already reported [18], which did not change significantly when combined with HDAC3 KO (Supplementary Fig. 2A). In line, protein levels of the cell cycle regulator p21 were not significantly modulated by HDAC3 KO alone in both cell lines while they rose after IR compared to non-irradiated ones with no further increase after HDAC3 loss-of-function (Supplementary Fig. 2B). Altogether, these data suggest that HDAC3 can support cell survival of FP-RMS after irradiation by protecting cells from IR-induced pro-apoptotic signals.

### Combination of HDAC3 depletion and IR impairs FP-RMS cells tumorigenic features in vitro and tumor growth in vivo

**Fig. 3:**
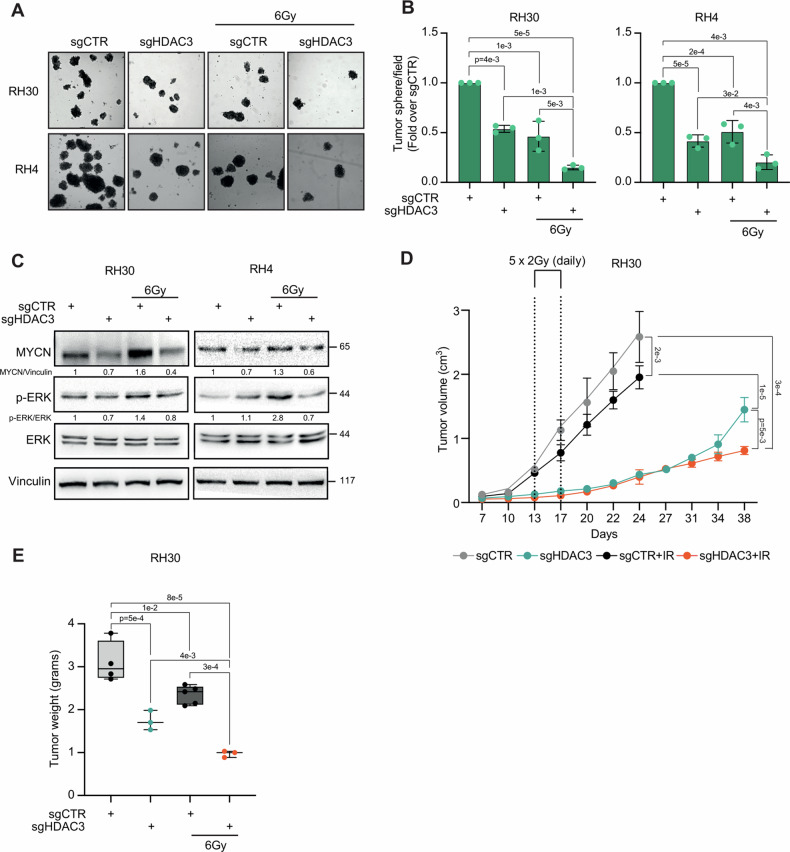
HDAC3 depletion in combination with IR reduces stemness and tumorigenicity in vitro and suppresses growth in vivo. **A** Representative light microscopy pictures of cancer stem cell formation assay on RH30 and RH4 cells infected with either sgControl (sgCTR) or sgRNA against HDAC3 (sgHDAC3) at 72 h post-infection and then treated or not with 6 Gy of IR. Scale bar = 50 μm. **B** Histogram depicts the quantification of rhabdospheres number per field. *n* = 3 independent experiments, data presented as mean values ± SD, one-way ANOVA. **C** Representative western blot (*n* = 3) of RH30 and RH4 cells treated as in (**A**) reporting MYCN, pERK and ERK protein levels. Vinculin was used as loading control. **D** Tumor volume of sgCTR (*n* = 9), sgHDAC3 (*n* = 6) of RH30 cells xenografts assessed by caliper measurement represented in mm^3^ followed for 48 days post-inoculation. 13 days after inoculation mice were irradiated each 3 days for 5 times with a single dose of 2 Gy of IR. Data presented as mean values ± SD, two-way ANOVA. **E** Tumor weight of transplanted RH30 cell xenografts treated as in (**D**). Data presented as mean values ± SD, two-way ANOVA.

Then, we evaluated the functional effects of HDAC3 depletion on the in vitro tumorigenic properties of FP-RMS cells. FP-RMS cells have shown sphere-forming ability when cultured in stemness medium (3D cultures) giving raise to rhabdospheres, i.e., cancer stem cells (CSCs) which express stemness markers and are highly tumorigenic and resistant to conventional chemotherapeutics [37]. The acquisition of stem-cell like characteristics of cancer cells has been associated with radioresistance [38, 39]. Therefore, we assessed the stem-related features of HDAC3-depleted FP-RMS cells by measuring their ability to form rhabdospheres in stemness culture conditions. To do so, 72 h after transduction, CRISPR/CAS9-mediated KO cells were subjected to IR (6 Gy) or left non-irradiated and 6 h later seeded in stemness medium. HDAC3 KO and IR alone greatly lowered the number of rhabdospheres 10 days post-seeding compared to non-irradiated sgCTR (Fig. 3A, B). Notably, when coupled with HDAC3 KO, IR impaired sphere-forming ability more significantly than what detected with either single HDAC3 depletion or IR alone.

As shown in Fig. 3C, IR increased MYCN protein levels, in agreement with our previous findings [18], and upregulated ERKs phosphorylation in FP-RMS cells. Moreover, HDAC3 depletion alone downregulated the basal levels of MYCN while did not modulate ERK activation in both cell lines. Notably, HDAC3 depletion counteracted the effects of IR on both MYCN, and ERKs^PO4^ accumulation in RH30 and RH4 cells restoring their expression to levels present in control cells. Then, we performed animal studies to examine the effects of HDAC3 KO in the presence and absence of IR in vivo. CD1 nude mice were subcutaneously injected with sgCTR or sgHDAC3 RH30 cells (10 mice each) and, after tumors reached a volume of ~0.3–0.5 cm^3^, a subset of animals was irradiated [18]. Interestingly, only 6 out of 10 mice injected with sgHDAC3 RH30 cells generated tumors suggesting that HDAC3 depletion alone could highly impact the in vivo tumorigenicity of FP-RMS cells. Radiation treatment was performed with a daily dose of 2 Gy for 5 consecutive days (Fig. 3D). When both sgCTR and sgCTR + IR mice reached the maximum measurable volume (about 2–2.5 cm^3^) they were sacrificed (Fig. 3D). At this time point, the tumor volume of sgHDAC3 masses were markedly reduced compared to control tumors, with no differences between irradiated and non-irradiated sgHDAC3 mice (Fig. 3D). At the end of the experiments, the volumes of sgHDAC3 tumors were still significantly lower than the corresponding controls sacrificed 14 days earlier, respectively by 46% for the non-irradiated group and by 58% for the irradiated ones (Fig. 3D). Moreover, tumor volumes of irradiated sgHDAC3 mice showed greater reduction in comparison with non-irradiated sgHDAC3 and irradiate control mice by further 45% and 58% respectively (Fig. 3D, E) suggesting additive effects of HDAC3 depletion and IR on tumor growth in vivo.

Altogether, these data demonstrated that the HDAC3 depletion radiosensitize FP-RMS cells strongly reducing their tumorigenic features in vitro and impairing tumor growth in vivo.

### HDAC3 KO promotes IR-induced Double-Strand Breaks (DSBs) and restrains the ability of FP-RMS to activate DNA Damage Response (DDR) and pro-survivor pathways

**Fig. 4:**
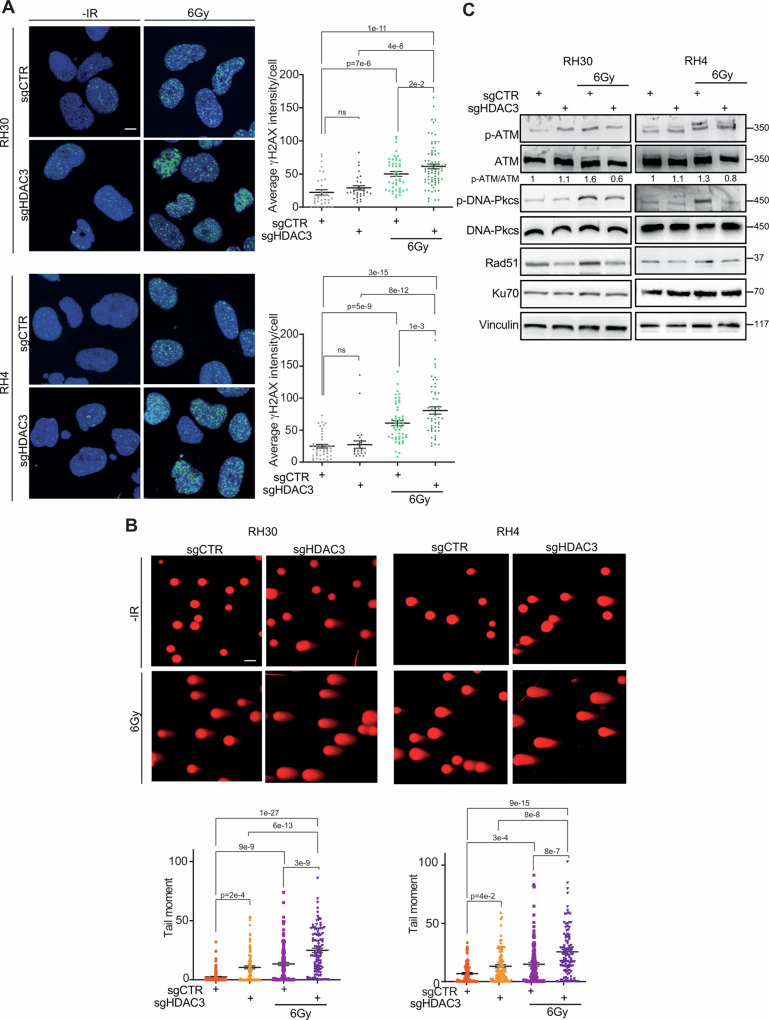
HDAC3 depletion in FP-RMS cell lines combined to IR induces DNA damage and impairs DNA repair. **A** Representative immunofluorescence images of γH2AX foci (green, left) (*n* = 3 independent biological replicates) in HDAC3 depleted -RH30 and -RH4 cells 6 h post IR with 6 Gy. DAPI (blue) was used as nuclear counterstain. Images were taken using confocal microscopy with a ×60 oil immersion objective lens. Scale Bar = 10 μm. Scatter plot of γH2AX average intensity per cell number (right) in HDAC3 depleted -RH30 and -RH4 cells after 6 h post IR with 6 Gy. Graph represents the mean of three independent experiments ±SEM, ANOVA one-way test. Exact *p*-values are reported in the figure. **B** Analysis of DSBs accumulation by the neutral Comet assay. Representative images are reported above. RH30 and RH4 HDAC3 KO cells were exposed or not to 6 Gy of IR and after 6 h subjected to the neutral Comet assay. In the scatter plot below, data are presented as mean tail moment ±SEM, ANOVA one-way test. Exact *p* values are reported in the figure. **C** Representative western blot (*n* = 3 independent biological replicates) depicting the effect of IR (6 Gy), HDAC3 depletion and their combination on DNA damage. The cells were processed 6 h post IR. pATM (Ser 1981), total ATM, pDNA-PKcs (Thr2609), total pDNA-PKcs, Rad51, and Ku70 protein levels were detected. All protein levels were normalized on Vinculin.

The main effect by which IR induces cancer cell death is the induction of DNA damage. Notably, tumor cells, usually resistant to IR, efficiently repair the DNA damage escaping from cell death and thereby promote cancer cell survival. Therefore, we evaluated the induction of DNA damage in HDAC3 depleted RH30 and RH4 cells 6 h post-6 Gy IR by analyzing immunofluorescence (IF) for γH2AX (H2AX phosphorylated on Ser139) foci [40]. IR alone significantly increased the γH2AX foci intensity in sgCTR RH30 and RH4 cells while HDAC3 KO did not enhanced the appearance of γH2AX foci compared to non-irradiated cells (*p* = 0.3 for RH30 and *p* = 0.7 for RH4 respectively) (Fig. 4A). However, a significant increase of γH2AX foci was detected in HDAC3-depleted irradiated cells compared to both irradiated sgCTR and non-irradiated sgHDAC3 cells in both cell lines (Fig. 4A). In addition, since IR preferentially induces DSBs instead of DNA single-strand break, we performed the neutral Comet assay by measuring the tail moment of cells: the assay is based on the detection of fragments of DNA strands that migrate from the nucleus forming a tail-like comet structure, thus allowing the determination of DNA DSBs at the single-cell level [41]. As shown in Fig. 4B, the analysis revealed that IR or HDAC3 depletion alone resulted in accumulation of DSBs in comparison with non-irradiated controls. Notably, the combination of IR and HDAC3 loss-of-function significantly increased DSBs compared to IR and HDAC3 depletion alone.

These results suggested that HDAC3 could be involved in the response to DNA damage. Thus, we performed a Pearson correlation analysis between *HDAC3* expression levels and drug sensitivity data from CTD^2 dataset (DepMap) and found that it significantly and positively correlates with the Area Under the Curve (AUC), a parameter used to evaluate drug response in a dose-response curve (the higher the AUC the lower the sensitivity), of a set of DNA damage-inducing drugs, among which Pevonedistat [42], Rigosertib [43], and Venetoclax [44] (Supplementary Fig. 3A, B).

The effects of HDAC3 KO on DDR were then investigated by assessing the levels of key players in both homologous recombination (HR) and non-homologous end joining (NHEJ) pathway. Interestingly, as shown in Fig. 4C, IR alone induced phosphorylation/activation of ATM, the upstream factor involved in the phosphorylation of H2AX on Ser139 residue to signal DSBs [45]. Notably, HDAC3 depletion alone did not modulate pATM/ATM but counteracted ATM activation induced by IR. Additionally, irradiated RH30 and RH4 cells showed increased phosphorylation levels of DNA-Pkcs, key player of NHEJ [45] (Fig. 4C). While DNA-Pkcs phosphorylation was not modulated by HDAC3 KO, HDAC3 depletion led to a notable reduction of the IR-induced DNA-Pkcs phosphorylation. Moreover, while IR treatment alone promoted upregulation of Rad51, a central HR component essential for the strand invasion during repair [45], HDAC3 depletion lowered its expression in comparison to non-irradiated control and neutralized the effects of IR restoring Rad51 basal levels (Fig. 4C). Interestingly, at mRNA levels we did not observe any significant change in the expression levels of genes involved in DNA repair after both HDAC3 KO and IR alone or in combination, suggesting post-transcriptional effects of both approaches on DNA repair pathways (Supplementary Fig. 3C, D).

Remarkably, there is no discernible impact on the expression of Ku70, an upstream effector of NHEJ involved in the end resection of DSBs [45], suggesting a more pronounced negative influence of HDAC3 depletion on HR pathway at least in part via Rad51 modulation.

Overall, these findings demonstrate that HDAC3 depletion impairs DNA repair in FP-RMS cells in response to IR-induced DNA damage.

### MC4448 is a new potent and selective inhibitor of HDAC3 that affects FP-RMS cells growth in vitro

**Fig. 5:**
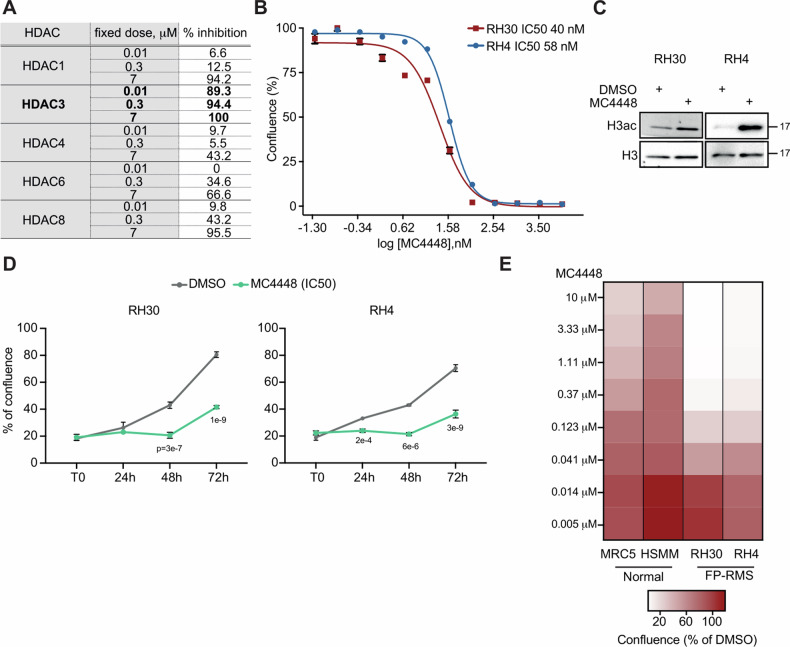
MC4448 is a new potent and highly selective HDAC3 inhibitor. **A** Biochemical data of MC4448 against HDAC1, −3, −4, −6 and −8 at three fixed doses (0.01, 0.3, and 7 µM). **B** Dose-response curves of RH30 and RH4 cells treated with increasing concentration of MC4448. Graph represents the mean of three independent experiment ±SEM. **C** Representative western blot (*n* = 3) of RH30 and RH4 treated with MC4448 IC50 for 24 h showing levels of acetylated H3. Vinculin was used as loading control. **D** Growth curve analysis of RH30 and RH4 cells treated as in (**C**). *n* = 3 independent experiments, data presented as mean values ± SD, Student’s two-tailed *t* test. **E** Heatmap depicting dose response effect of MC4448 treatment for 72 h on FP-RMS cells (RH30 and RH4), and Normal Human Skeletal Muscle Myoblast (HSMM) and Normal Fetal Human Lung Fibroblast (MRC5).

To date, class-I HDACs inhibitors used in clinical trial (such as Entinostat and Romidepsin) showed collateral toxicity mainly due, at least in part, to non-isoform specific inhibition joined to off-target effects [20]. Thus, we focused on the development of a new chemical entity designed to selectively inhibit HDAC3, with the aim to mitigate toxicity maintaining unaltered the anti-tumor effectiveness. Starting from the structure of resminostat, a pan-HDACi in clinical trials alone and in combination for solid cancer treatment [46] we applied several chemical manipulations to prepare a library of analogs to be tested against various HDAC isoforms. Among them, MC4448 emerged as a highly potent and selective HDAC3 inhibitor, displaying subnanomolar inhibition towards HDAC3 and over 3 to 5 magnitude orders of selectivity towards the other tested isoforms. In detail, MC4448 was screened against human recombinant HDAC1, −3, −4, −6, and −8, in 10-dose mode with 3-fold serial dilution starting from 200 μM solution to determine its inhibitory potency. The fluorogenic monoacetylated peptide from p53 residues 379–382 (Arg-His-Lys-Lys(Ac)AMC) was used to detect inhibitory activity against HDAC1, −3, −6, while for HDAC8 the diacetylated peptide from p53 residues 379–382 (Arg-His-Lys(Ac)-Lys(Ac)AMC) was used. For HDAC4 (class IIa HDACs), the fluorogenic class IIa (Boc-Lys(trifluoroacetyl)-AMC) substrate was employed [47]. Figure 5A shows values of % inhibition by MC4448 at three fixed doses: 0.01, 0.3, and 7 µM, against HDAC1, −3, −4, −6, and −8, to highlights its huge anti-HDAC3 potency and selectivity.

To test the efficacy of MC4448 in inhibiting HDAC3 activity in FP-RMS cells, we performed a dose-response curve treating RH30 and RH4 cells with escalating doses of the drug for 72 h (Fig. 5B). Both cell lines showed IC_50_ in a nanomolar range (Fig. 5B). Treatment of tumor cells with the corresponding MC4448 IC_50_ (40 nM for RH30 and 58 nM for RH4) for 24 h resulted in increased levels of histone H3 acetylation, confirming inhibition of the major HDACs target de-acetylation (Fig. 5C). Moreover, we performed a growth curve assay on RH30 and RH4 cells treated with MC4448 for 72 h. The growth curve analysis revealed a significant inhibitory effect of MC4448 on the proliferation of both cell lines compared to the DMSO-treated control cells starting 48 h post-treatment (Fig. 5D). To assess possible toxic effects of MC4448 on healthy tissues, we treated normal cells such as human lung fibroblast (MRC5) and myoblasts (HSMM) with escalating doses of the agent from 5 nM to 10 μM. As shown in Fig. 5E, both MRC5 and HSMM showed marked decrease in sensitivity to MC4448 compared to FP-RMS cells, both at high and low doses. Altogether, these data suggest that MC4448 is a new potent and highly tumor-specific HDAC3 inhibitor that hampers FP-RMS tumor cell survival at nM doses without affecting normal cells.

### MC4448 mimics HDAC3 genetic loss-of-function effects in radiosensitizing FP-RMS cells in vitro by inducing Caspase 3/7 dependent apoptosis and hampering tumorigenic features

**Fig. 6:**
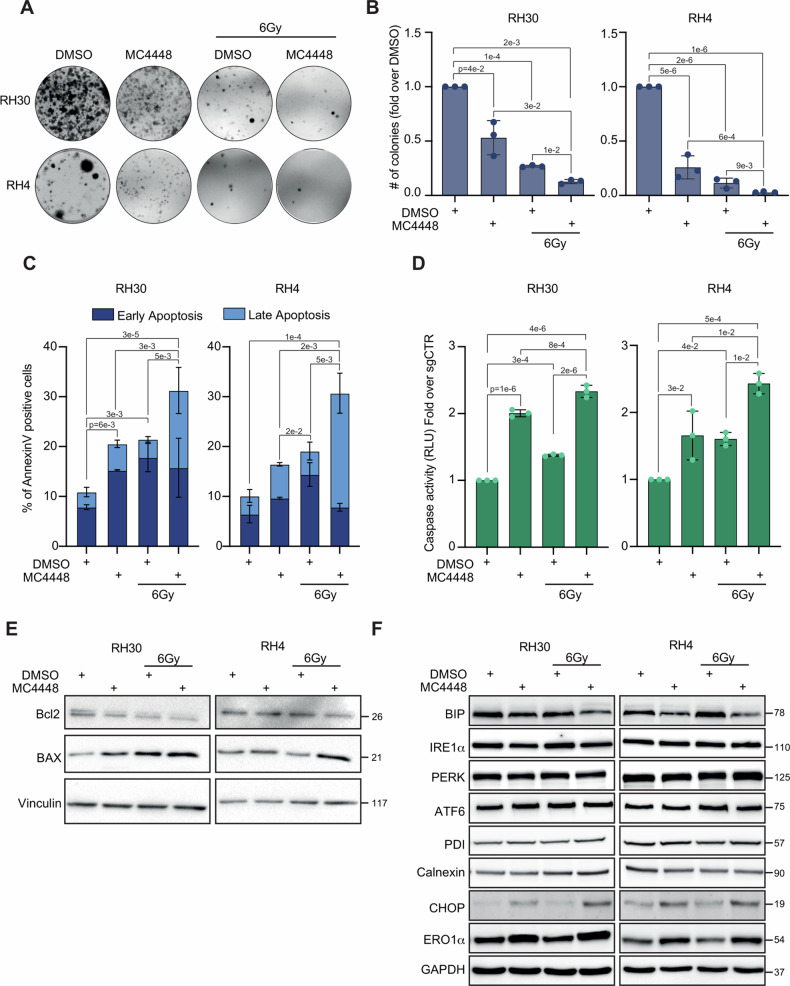
MC4448 treatment mirrors HDAC3 depletion effects radiosensitizing FP-RMS cells. **A** Representative images of RH30 and RH4 colonies stained with crystal violet 12 days post seeding. FP-RMS cells were treated with either MC4448 IC50 (40 nM for RH30 and 58 nM for RH4) or DMSO for 24 h and then irradiated or not with 6 Gy. The cells were processed 6 h post IR. **B** Histograms depicting the plating efficiency of RH30 and RH4 treated as in (**A**). Graph represents the mean of three independent experiments ±SD, one-way ANOVA. Exact *p* values are reported in the figure. **C** Histogram depicting the percentage of Annexin-V positive/7-AAD single- and double-positive RH30 and RH4 cells treated as in (**A**). Graphs represent the mean of three independent experiments ±SD, one-way ANOVA. Exact *p* values are reported in the figure. **D** Histogram depicts the quantification of Caspase 3/7 activity in RH30 and RH4 cells treated as in (**A**). Graphs represent the mean of three independent experiments ±SD, one-way ANOVA. Exact *p*-values are reported in the figure. **E**. Representative western blot (*n* = 3) depicting levels of Bcl2 and BAX of RH30 and RH4 cells treated with either DMSO or MC4448 IC50 for 24 h and then treated or not with 6 Gy of IR. Vinculin was used as loading control. **F** Representative western blot (*n* = 3) depicting levels of BIP, IRE1α, PERK, ATF6, PDI, Calnexin, CHOP, and ERO1α of RH30 and RH4 cells infected with either DMSO or MC4448 at for 24 h and then treated or not with 6 Gy of IR. GAPDH was used as loading control.

We evaluated the radiosensitizing potential of MC4448 by assessing colony formation efficiency of RH30 and RH4 cells following IR exposure. Cells were pre-treated with MC4448 or vehicle (DMSO) for 24 h and then irradiated with 6 Gy or left non-irradiated before seeding for the clonogenic assay 6 h later (Fig. 6A, B). MC4448, when administered as a single agent, reduced the colony-forming ability of both cell lines although to a lower extent than IR alone in comparison to non-irradiated vehicle-treated cells (Fig. 6B). Strikingly, MC4448 significantly enhanced the radiation-induced cytotoxicity in both cell lines, resulting in an almost complete suppression of colony formation in RH4 cells (Fig. 6B). These data are in accordance with the results obtained in RH30 and RH4 cells with HDAC3 KO following IR. To functionally assess the impact of MC4448 treatment on the response of FP-RMS cells to IR, we evaluated the induction of apoptosis by performing Annexin V staining on RH30 and RH4 cells pre-treated with MC4448 for 24 h, then irradiated or not, and cultured in the presence of the compound for the next 24 h. As depicted in Fig. 6C, MC4448 and IR as single treatments augmented the Annexin V positivity in both cell lines *vs* vehicle non-irradiated cells although they did not reach the significance in RH4 cells. Moreover, irradiated MC4448-treated RH30 and RH4 cells exhibited a significant increase of the apoptotic cell percentage compared to their non-irradiated counterparts (Fig. 6C). Interestingly, an increase of the late apoptotic population, as opposed to the early apoptotic fraction, was observed in both cell lines (Fig. 6C). The apoptotic phenomenon was paralleled by an upregulation of the activity of the apoptosis-associated marker Caspase 3/7 in FP-RMS cells treated with either MC4448 or IR compared to non-irradiated vehicle ones (Fig. 6D). Consistent with the heightened Annexin V-positivity, the co-treatment with MC4448 for 24 h and IR led to a sustained upregulation of the activity of Caspase 3/7 (Fig. 6D), downregulated Bcl2 and upregulated BAX (Fig. 6E), with respect to single treatments. Moreover, as described after the genetic KO of HDAC3, MC4448 triggered the activation of ER stress-related pro-apoptotic UPR pathways by downregulating the protein expression levels of BIP, and upregulating the CHOP/ERO1α axis with no effects on IRE1α, PERK, ATF6, PDI, and calnexin (Fig. 6F). Furthermore, we detected an increase in the LC3B-II/LC3B-I ratio, and p62, markers related to autophagy, suggesting an increase in autophagosome assembly, with a contemporarily block of degradation (Supplementary Fig. 4A) [48, 49].

Thus, MC4448 could potentiates the cytotoxic effects of IR by sustaining partly the pro-apoptotic ER stress activation, modulating the expression of apoptotic regulators in favor of pro-apoptotic ones, and finally inducing the activation of Caspase3/7-mediated apoptotic cell death.

**Fig. 7:**
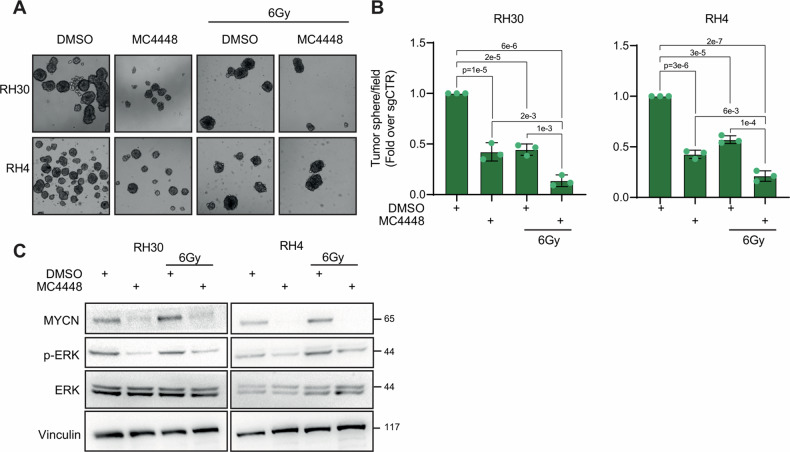
MC4448 treatment in combination with IR reduces stemness and counteracts IR-dependent induction of MYCN and p-ERK. **A** Representative light microscopy pictures of cancer stem cell formation assay on RH30 and RH4 cells treated with either DMSO or MC4448 IC50 for 24 h and then treated or not with 6 Gy of IR and grown for 2 weeks. Scale bar = 50 μm. **B** Histogram depicts the quantification of rhabdosphere numbers per field. *n* = 3 independent experiments, data presented as mean values ± SD, one-way ANOVA. **C** Representative western blot (*n* = 3) of RH30 and RH4 cells treated as in A reporting MYCN, pERK and ERK protein levels. Vinculin was used as loading control.

As observed for HDAC3 KO cells, while IR alone induced G2/M cell accumulation we did not detect any modulation of cell cycle distribution neither in MC4448-treated cells nor in cells co-treated with MC4448 and IR (Supplementary Fig. 4A, B). However, again in agreement with the results obtained in HDAC3 KO, p21 protein levels increased in RH30 and RH4 cells subjected to MC4448 and IR single treatments while no significant additive effects were evident in the combinatorial treatment (Supplementary Fig. 4C). Furthermore, MC4448 and IR alone critically dropped the number of rhabdospheres 10 days post-seeding in stemness culture medium compared to vehicle-treated cells with a higher decrease *vs* each single approach when treatments were combined (Fig. 7A, B), as observed in sgHDAC3 depleted cells. In addition, MC4448 treatment downregulated the basal levels of MYCN and ERKs^PO4^ and overcome the IR-induced MYCN and ERKs^PO4^ accumulation in RH30 and RH4 cells (Fig. 7C). With the attempt to dissect the molecular mechanism responsible for the radiosensitizing effects of HDAC3 inhibition, we analyzed the role of ERKs by inhibiting them using the MEK/ERKs inhibitor U0126. MC4448 mirrored the effects of HDAC3 depletion reducing colony formation when applied alone and further impairing both processes in IR-treated cells compared to single approaches (Supplementary Fig. 5A–C). Inhibiting ERKs with U0126 alone did not decrease cell colonies number vs vehicle but markedly affected it in irradiated cells suggesting the pathway important for the response to IR even in FP-RMS [39, 48] (Supplementary Fig. 5A–C). Moreover, U0126 improved the MC4448-induced radiosensitization further reducing colony formation compared to each single agent + IR (Supplementary Fig. 5A–C).

As a model to activate ERKs, we inhibited p38 MAPK (hereafter p38) with SB203580, which we previously showed to upregulate ERKs phosphorylation and induce radioresistance in FN-RMS cells [39]^.^ Treatment with SB203580 alone had no or mild effect on colony formation in untreated and IR-treated cells, respectively, and did not affect the ERKs activation in response to IR (Supplementary Fig. 5A, D, E). Notably, SB203580 was able to counteract colony formation reduction due to MC4448 and to increase the intrinsic resistance to IR of FP-RMS cells when applied in combination with the HDAC3i (Supplementary Fig. 5A, D). These effects were associated to restoration of ERKs phosphorylation in SB203580 + MC4448 cells (Supplementary Fig. 5E). Altogether, these findings suggest that HDAC3 promotes resistance to IR partly by maintaining ERKs activated.

Since HDAC3 KO overcome JNK phosphorylation/activation due to IR, we investigated the effects of MC4448 in combination with the JNKi SP600125 on IR-induced JNKs activation. As shown in Supplementary Fig. 5A, F, SP600125 as single agent decreased the number of colonies compared to vehicle and strongly hampered colony formation of irradiated cells with no further effects when used together with MC4448.

HDAC3i mildly affected the phosphorylation of JNK and of its direct target c-Jun under basal conditions but was able to overcome JNK activation after IR (Supplementary Fig. 5G). According to the functional effects, SP600125 as single agent reduced phosphorylated JNK and c-Jun below the control levels and maintained their activation low alone or in combination with MC4448 after IR (Supplementary Fig. 5G).

Altogether these data demonstrate that MC4448 mirrors the radiosensitizing effects observed in HDAC3-depleted FP-RMS cells. Moreover, they also suggest a critical role for HDAC3 in supporting resistance to irradiation at least in part regulating ERKs and JNKs phosphorylation/activation.

### MC4448 treatment increases IR-induced DSBs and inhibits DNA repair pathways

**Fig. 8:**
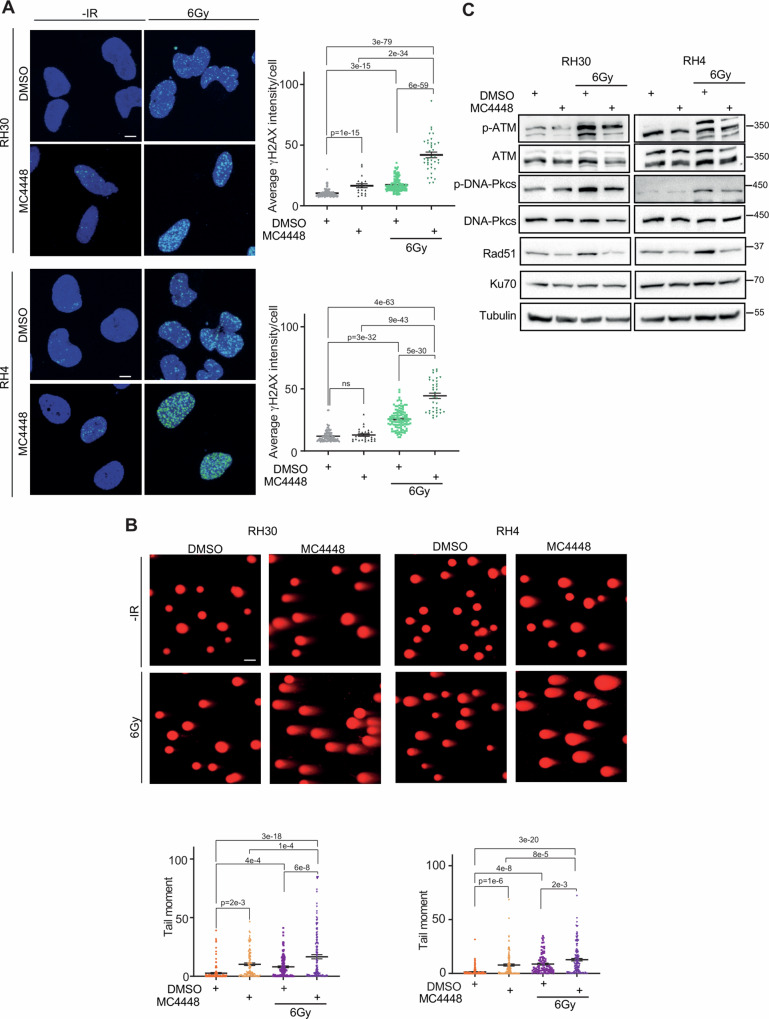
The combination of IR and MC4448 inhibitor induces DNA damage and impairs DNA repair. **A** Representative immunofluorescence images of γH2AX (green, left) (*n* = 3 independent biological replicates) in MC4448 treated RH30 and RH4 cells 6 h post IR with 6 Gy. DAPI (blue) was used as nuclear counterstain. Scale Bar = 10 μm. Scatter plot of γH2AX average intensity per cell number (right) in MC4448 treated RH30 and RH4 cells after 6 h post IR with 6 Gy. Graph represents the mean of three independent experiments ±SEM, ANOVA one-way test. Exact *p* values are reported in the figure. **B** Analysis of DSBs accumulation by the neutral Comet assay. Representative images are reported above. RH30 and RH4 cells were treated with MC4448 and then exposed to 6 Gy of IR. After 6 h FP-RMS cells were subjected to the neutral Comet assay. In the scatter plot below, data are presented as mean tail moment ± SEM, ANOVA one-way test. Exact *p* values are reported in the figure. **C** Representative western blot (*n* = 2 independent biological replicates) of FP-RMS cells treated with MC4448 and IR (6 Gy). The cells were processed 6 h post IR to evaluate the effect of combination of treatments on DNA damage response. pATM (Ser 1981), total ATM, pDNA-PKcs (Thr2609), total pDNA-PKcs, Rad51, and Ku70 protein levels were detected. All protein levels were normalized on Vinculin.

To further confirm the ability of MC4448 treatment in phenocopying the HDAC3 KO effects after IR, we examined the presence of DNA damage in irradiated MC4448-treated RH30 and RH4 cells, analyzing H2AX phosphorylation on Ser139 (γH2AX) by immunofluorescence, as described for HDAC3 KO cells. Figure 8A showed that both IR and MC4448 treatment alone increased the intensity of γH2AX foci in RH30 cells compared to non-irradiated vehicle cells while this effect was evident only for IR in RH4 ones (*p* = 0.7 RH4 DMSOvsMC4448). However, the combination of MC4448 and IR further and significantly increased γH2AX foci intensity compared to single treatments (Fig. 8A). Moreover, as depicted in Fig. 8B, the neutral comet assay showed that both MC4448 treatment and IR alone led to a modest, even if significant, accumulation of DSBs compared to non-irradiated control. Also in this case, the combination of IR and MC4448 markedly increased DSBs compared to each single treatment.

Then, to explore the effects of MC4448 treatment on DDR, we assessed the levels of key players in both HR and NHEJ pathways. Figure 8C reveals that IR, as a standalone treatment, induced ATM and DNA-Pkcs phosphorylation/activation associated to Rad51 protein levels upregulation. HDAC3 pharmacologic inhibition alone, instead, did not modulate basal ATM or DNA-Pkcs activation but down-regulated Rad51 protein expression (Fig. 8C). Moreover, the combination of MC4448 and IR resulted in a notable reduction of IR-induced ATM and DNA-Pkcs phosphorylation and Rad51 overexpression (Fig. 8C), suggesting impaired DNA repair. Intriguingly, at the mRNA level no modulation was observed in genes involved in DNA repair, suggesting once more an indirect effect of HDAC3 on DNA repair pathways (Supplementary Fig. 6A, B). The evidence herein collected suggests that MC4448 acts as a radiosensitizer agent in FP-RMS RH30 and RH4 cells by preferentially impairing the activation of HR-DSBs in both RH4 and RH30 cells, supporting the ability of HDAC3 to promote DNA repair.

## Discussion

First line therapy for high-risk RMS such as PAX3-FOXO1 FP-RMS includes RT, chemotherapy, and surgical removal. Although recent improvements in RMS patient outcomes are linked to the use of RT [10], approximately one third of patients with localized disease experiences local recurrence after IR, suggesting the need for radiosensitizing strategies. We have recently shown that class-I HDACi Entinostat [18] and Romidepsin [19] radiosensitize FP-RMS cells in vitro and in vivo. However, despite promising preclinical studies, pan- and class-selective HDACi showed only modest success as in monotherapy in human clinical trials on solid tumors, frequently showing off-target related toxicities [49]. Therefore, the identification of selective HDACs that can be targeted to promote the response to RT is of great importance in FP-RMS. In this study, we identify HDAC3, a class-I HDAC isoform, as a specific target for enhancing radiosensitivity in FP-RMS cells in vitro and in vivo.

Analysis performed on RMS patients’ samples, PDX and cell lines, showed that all class-I HDACs were overexpressed compared to their normal counterparts, regardless of the gene fusion status. Moreover, among all class-I HDACs, RMS cell lines exhibit the highest dependency on HDAC3 expression. This is in line with our previous results demonstrating that HDAC3 is needed for FN-RMS and FP-RMS in vitro cells proliferation [29, 50]. Furthermore, RMS cell lines resulted one of the most HDAC3-dependent tumor cell lines among several other tumor cell types. Notably, in this scenario, class-I HDACs siRNA screening on FN-RMS and FP-RMS cell lines indicated HDAC3 as the only isoform affecting the FP-RMS subtype cell ability to survive forming colonies. Moreover, in FP-RMS but not FN-RMS only the expression of HDAC3 correlated with a poor prognosis, suggesting HDAC3 as candidate marker of responsiveness to RT.

These results are in line with a recent study involving HDAC3-indirect regulation of PAX3-FOXO1 levels seen after Entinostat treatment, which promotes response to irradiation in FP-RMS models [22].

Interestingly, we did not observe radiosensitivity in the FP-RMS cells silenced for the other class-I HDACs isoforms. This could depend on the extent of functional redundancy among the HDACs, as already demonstrated between HDAC1 and HDAC2. HDAC3, by contrast, shows unique and not compensable structural variations [50]. Moreover, the lack of radiosensitizing effects after HDAC3 silencing in FN-RMS cells could be ascribed, at least in part, to the induction of myogenic differentiation after HDAC3 depletion more efficiently in FN-RMS than FP-RMS cells as we showed previously [29] that could result in differentiating cells notoriously more resistant to IR [51]. Therefore, we knocked out HDAC3 in FP-RMS cells by a CRISPR/Cas9-mediated strategy using a dual-sgRNAs-expressing vector to increase gene targeting [29]. Functionally, our results demonstrate that the HDAC3 depletion amplifies the response to IR in vitro further reducing FP-RMS cells clonogenic ability, inducing apoptotic cell death, and impairing stemness. Moreover, HDAC3 KO also hampers tumor growth in vivo in the absence of and more efficiently after IR.

IR preferentially induces cell death by apoptosis [32]. Notably, despite it has been demonstrated that HDACs preserve cancer cells from IR-induced apoptosis [52], no evidence on the direct role of HDAC3 has been reported so far. Herein, we found that, HDAC3 depletion more efficiently increased PARP1- and caspase-dependent apoptosis in irradiated than in non-irradiated cells suggesting that FP-RMS cells need HDAC3 to survive after IR. This agrees with the ability of HDAC3 to restrain apoptosis induced by several stimuli in different cancer cell types. Furthermore, although HDAC3 KO in the absence of IR and IR alone are unable to induce apoptosis at the examined time points compared to the non-irradiated control, both treatments alone drastically reduce the clonogenic activity in FP-RMS cells. Moreover, HDAC3 reduction alone also results in caspase activation. These findings suggest that the combination of HDAC3 depletion with IR induces cell death as the completion of a process already partially triggered by each individual treatment.

With the aim to identify the molecular mechanisms underlying the pro-death effects of HDAC3 loss-of-function in the IR context, we focused on DNA damage and DSBs, which are the main cause of post-IR cell death [53].

Herein, differently to what described in liver cancer cells [54], HDAC3 depletion alone is ineffective in inducing phosphorylation of H2AX (i.e., γH2AX), a marker of DNA damage [55], but highly enhances IR-induced γH2AX increase. However, HDAC3 KO and IR alone modestly but significantly induce DSBs compared to non-irradiated control cells, which is markedly augmented by HDAC3 KO/IR combination. Accordingly, it has been reported that HDACs, by compacting chromatin, make DNA less susceptible to DSBs [56], with HDAC3 for the maintenance of chromatin structure and genome stability [57].

However, despite the accumulation of DNA damage in irradiated cells HDAC3-depleted could explain the extraordinary susceptibility of these cells to the activation of death programs, justifying the increase in apoptosis previously described, cancer cells possess the ability to inappropriately activate DDR mechanisms mediated through the activation of HR and/or NHEJ pathways [58], finally leading to cells survival. Moreover, HDACs have been shown to sustain DDR [16], and cancer cells surviving can accumulate new mutations responsible of a more aggressive and radioresistant phenotype [56].

We show here that, while HDAC3 depletion per se is unable to affect the basal activation of ATM and DNA-Pkcs, which are upstream DDR signaling proteins of HR and NHEJ pathways, respectively, it clearly blocks their IR-induced activation. Moreover, HDAC3 depletion decreases the protein basal levels and the IR-induced upregulation of Rad51, an essential factor for strand invasion during HR, whilst it does not affect the levels of Ku70, which regulates the end resection of DBSs during NHEJ [45]. Therefore, whilst NHEJ pathway results to be not completely turned off by HDAC3 depletion, the effects seem to be total for HR pathway.

It has been shown that HDACs protect from DSBs-induced apoptosis by sustaining the expression of Rad51 [59], and that the cleavage of Rad51 by a caspase-dependent mechanism mainly contributes to the cell death response induced by IR-mediated DNA damage [60]. Therefore, we hypothesize that HDAC3 depletion limits DSB repair by promoting caspase-mediated degradation of Rad51. Notably, Rad51 has been shown to be a functional biomarker for HR deficiency in cancer condition known to confer a therapeutic vulnerability in cancer [61]. Thus, targeting HDAC3 to impair the ability of FP-RMS cells to activate HR pathway after DNA damage could represents a critical strategy to elicit the radiotherapeutic vulnerability in this cancer type. Overall, the HDAC3 depletion-induced attenuation of ATM and DNA-Pkcs activation, coupled with the suppression of Rad51 expression, collectively underscores the intricate regulatory effect of HDAC3 on DNA repair mechanisms in FP-RMS in response to IR, particularly emphasizing a significant impact on HR pathway.

The fact that NHEJ is not completely inhibited does not exclude its key role. It has been shown that there is a functional interplay between DDR pathways [62]. Thus, we cannot exclude that the restrained activation of NHEJ after IR in HDAC3 KO cells can participate in further reducing HR activity, as well as that the restrained activation of HR can affect NHEJ bypassing failure in modulating Ku70.

DSBs occur directly or indirectly, because of Reactive Oxygene Species (ROS) accumulation [53]. ER stress induced by IR, functionally mediated by the activation of unfolded protein response cascade (UPR), can preserve from IR-induced unfolded/misfolded protein accumulation or induce ROS production [32]. Thus, depending on the duration and intensity of the stimulus, UPR can switch from pro-survival to pro-apoptosis signaling [63]. The accumulation of misfolded/unfolded proteins in the ER lumen frees PERK, IRE1α, and ATF6, the three effectors of UPR, from the inhibitory binding of BIP, the primary sensor and negative regulator of UPR cascade. PERK, IRE1α, and ATF6 then restore the ER homeostasis, by increasing protein folding, attenuating translation, and degrading mRNA. However, under high level and persistent stress, these signaling destined the cells to apoptosis by inducing the expression of CHOP transcription factor [63]. Herein, HDAC3 depletion downregulates the expression of BIP, and upregulates the expression of CHOP and its pro-oxidant effector ERO1α [63, 64]. It has been shown that BIP sustains FP-RMS aggressiveness [65], and that the basal activation of UPR cascade promotes survival of RMS cells, whilst its robust activation induces apoptosis [66]. Moreover, CHOP/ERO1α axis induces apoptosis by inducing BAX and retraining Bcl2 expression, accordingly with their modulation herein described after depleting HDAC3 [64]. Interestingly, the pro-survival effectors of UPR, calnexin [33, 34] and PDI [35, 36] are basally expressed by FP-RMS suggesting that in the absence of cytotoxic stimuli these could sustain cytoprotective signals. Notably, it has been shown that caspase-3 or caspase-7 cleave calnexin, whose cleaved product leads to the attenuation of apoptosis [67]. Therefore, we suggest that in FP-RMS, HDAC3 supporting BIP expression maintains a level of basal UPR activation, by sustaining calnexin and PDI expression, sufficient to ensure endogenous proteostasis and to prevent the accumulation of damaged proteins from triggering cytotoxic signals. Thus, the HDAC3/BIP axis would support the intrinsic radioresistance of FP-RMS.

The aberrant cell cycle redistribution is another critical molecular mechanism inducing radioresistance in cancer [68]. Indeed, irradiated tumor cells arrest the cell cycle progression to self-repair sublethal damaged DNA. Usually, fractionated RT induces arrest in the G2/M phase, the most radiosensitive phase of the cell cycle [69], thus allowing subsequent fractions to act more effectively. Accordingly, to our previously collected evidence [18], herein 6 Gy dose of X-rays blocks FP-RMS cells in G2/M, with no differences between HDAC3 KO or control cells. Thus, despite HDAC3 has been shown to be an essential cell cycle regulator [70], no differences were detected between irradiated HDAC3 KO or parental cell lines. Thus, the radiosensitization induced by targeting HDAC3 seems to be independent by cell cycle distribution.

CSCs have been identified in many tumor types and are the most malignant cells within a tumor [71]. A significant aspect of cancer management is the effect of radiotherapy (RT) strategies on CSCs. CSCs are a subset of tumor cells that divide slowly and possess self-renewal capabilities, playing a crucial role in tumor persistence, metastasis, and resistance to standard treatments in various cancer types. Recent findings indicate that CSCs in several tumors, including RMS, can resist ionizing radiation due to their unique metabolic state. This state is linked to elevated expression of genes and pathways associated with stem-like properties, activated DNA repair mechanisms, and modified levels of free radical scavengers [14]. IR preferentially kills non-CSCs or reprograms cancer cells to CSCs, which are a subpopulation of driving tumor cells, intrinsically radioresistant, and one of the major determinants of post-RT relapse [38, 39]. We show that HDAC3 depletion alone, and more efficiently in combination with IR, hampers the stemness ability of FP-RMS cells to grow as rhabdospheres. The effect of HDACs on chromatin condensation has been associated with the regulation and maintenance of CSCs phenotype [72], and we have recently shown that targeting Class I HDACs affects CSCs population [18, 19], accordingly with the key role of HDAC3 in this context [68, 73]. Notably, UPR has been identified to regulate cancer stemness UPR [74] which, given the role previously discussed, could play a key role in protecting FP-RMS CSCs from IR-induced death. Thus, HDAC3 seems to promote FP-RMS radioresistance partly by preserving the cancer cells population from IR-induced apoptosis by promoting cancer stemness.

We also demonstrated that HDAC3 depletion decreased basal MYCN protein levels and can counteract MYCN upregulation in irradiated cells. MYCN is a crucial driver and a survival gene needed for the maintenance of the tumorigenic phenotype in FP-RMS cells [75, 76]. Thus, the dramatic inhibitory effect on the survival potential and stemness of irradiated-HDAC3 KO cells could be partly ascribed to the inhibitory effect of HDAC3 depletion on MYCN expression. We previously showed reduced expression of MYCN following HDAC3 pharmacologic inhibition in FP-RMS cells [50]. Moreover, MYCN is induced by IR and linked to radioresistance in neuroblastoma [77]. Altogether, this data suggests a potential involvement of this transcription factor in the radioresistant phenotype of FP-RMS, which should be investigated in future.

Moreover, HDAC3 depletion also counteracts the IR-dependent induction of ERKs and JNKs phosphorylation/activation status, both known to collaborate in sustaining cancer survival, stem-like population and radioresistance of several cancer types [78–83], including in RMS [39, 84]. Notably, ERKs and JNKs signaling have been shown to differently protect against apoptosis following ER stress [85, 86]. Moreover, it has been shown that ERKs signaling pathway upregulates JNKs sustaining the aggressiveness of melanoma cells [87]. Therefore, we hypothesize that there may be an interplay between the phosphorylation/activation of ERK and JNK and that this ultimately determines cytoprotective effects by preserving from excessive/prolonged ER stress, and therefore acting as a mechanism of radioresistance. Thus, the lack of phosphorylation/activation of ERKs and JNKs in irradiated HDAC3 KO cells could increase the radiosensitivity of these cells.

Interestingly, HDAC3 depletion per se did not affect ERKs and JNKs phosphorylation. This may potentially be due to the evidence that HDAC3 activates ERKs particularly under the action of transforming growth factor β (TGF-β), which is absent or low expressed in basal conditions but is released by cells following radiotherapy to promote radioresistance [88, 89]. Although there is no direct evidence, the same mechanism could concern JNK, given the ability of TGF-β to induce JNKs activation [90].

We also unveil a role of HDAC3 in the in vivo tumor growth of FP-RMS cells showing that HDAC3 depletion strongly affects both the onset and growth of tumor masses. Indeed, only 6 out 10 mice subcutaneously xenografted with HDAC3-depleted RH30 cells developed palpable tumors. However, after a period of low tumor growth for those HDAC3 KO established tumors, they appear to regrowth suggesting that HDAC3 expression per se could be essential particularly for the in vivo engraftment and the first phases of tumor growth. However, HDAC3 loss-of-function radiosensitizes FP-RMS cells in vivo, allowing IR to maintain more efficiently low the tumor growth in tumors HDAC3-depleted when compared to HDAC3 KO alone.

In agreement, our analysis of the drug sensitivity dataset CTD^2 showed that HDAC3 expression in RMS cells correlates with the AUC, a parameter used to evaluate drug response (the higher the AUC the lower the sensitivity), of investigational DNA damage-inducing drugs such as Pevonedistat [42], Rigosertib [43] and Venetoclax [44], suggesting a potential involvement of this HDAC in the resistance to these drugs.

Considering these promising results, we have developed MC4448, a novel highly tumor-selective HDAC3-specific inhibitor, active towards HDAC3 at submicromolar level and displaying 3 to 5 magnitude orders selectivity for HDAC3 respect to the other tested HDAC isoforms. MC4448 is effective in reducing in vitro cell survival in FP-RMS cells and in increasing H3 acetylation at very low concentrations showing IC_50_ values in a nanomolar range. We demonstrate that the compound exhibits tumor-selectivity since the effect on normal human fibroblast and myoblast is negligible also at high concentrations. Furthermore, MC4448 treatment combined with IR closely recapitulates what observed with HDAC3 depletion, highlighting the specificity of our drug in targeting HDAC3. Indeed, MC4448 impairs FP-RMS cells clonogenicity and their ability to give rise to CSCs, induces caspase activation and downregulates MYCN, ERKs^PO4^ and JNKs^PO4^ protein levels. A confirmation of the importance of ERK inactivation in the MC4448 radiosensitization effects is given by the enhanced MC4448 radiosensitization effects of FP-RMS treated with ERKi, U0126. Furthermore, p38 inhibition, a model of ERK activation, was able to counteract the MC4448 radiosensitization effects. These effects could be due to restoration of ERKs phosphorylation in SB203580 treated cells. Altogether, these findings suggest that HDAC3 promotes resistance to IR partly by sustaining ERKs activation.

Interestingly, while HDAC3 inhibition and genetic depletion is able to counteract IR dependent JNK phosphorylation, SP600125 treatment is not able to enhance MC4448 radiosensitization effects. Conversly, JNK inhibition is able to sensitize FP-RMS to IR suggesting that it could be another promising radiosensitization strategy as already reported in other tumor types [30, 31].

In addition to greatly amplify these effects, the combination of MC4448 and IR also resulted in marked activation of pro-apoptotic ER-stress, apoptosis, and DDR inhibition. Notably, autophagy, a degradative process involved in removal damaged or unnecessary cellular components, implicated in many biological processes during cell survival and death [91], and shown to interact with ER stress in other cellular models [92], result blocked by MC448. In particular, although the increase in the LC3B-II/LC3B-I ratio indicates that the drug activates autophagy by inducing the formation of autophagosomes, the lack of descent of p62 indicates that these are not degraded [93]. This can result either from the lack of fusion of autophagosomes with lysosomes or from the lack of acidification of the autophagosomes. Future investigations will be done to understand the reason and functional significance of MC448-induced autophagy blockade. Of note, the increased late apoptotic cell percentage in MC4448/IR cells compared to what observed for HDAC3-depleted/IR ones, which causes accumulation of early apoptotic cells, seems to suggest that the inhibitory effect on HDAC3 activity by the compound occurs at earlier time points.

Altogether, our data unveil a novel role for HDAC3 in fostering radioresistance in FP-RMS cells partly by promoting DNA repair. They also suggest that pharmacologic HDAC3 inhibition is feasible in FP-RMS and could be also explored in combination with other DNA damage inducing approaches.

## Materials and Methods

### Bioinformatic analyses

Expression profiles of Class I HDACs in RMS patients and in normal skeletal muscle tissue were obtained using GSE108022 dataset available at Gene Expression Omnibus repository, analysed with iDEP 1.1 (http://bioinformatics.sdstate.edu/idep96/) and plotted with GraphPad Prism 8. Gene and protein expression in RMS PDX was obtained from St.Jude Cloud (https://viz.stjude.cloud/st-jude-childrens-research-hospital/visualization/epigenetic-landscape-of-rhabdomyosarcoma-subtypes~67). Gene expression data from normal human fibroblast and RMS cell lines was obtained from DepMap using the 22Q2 public dataset for gene expression. Data from CRISPR genome screening on 11 RMS cell lines were downloaded from DepMap (https://depmap.org/portal/). Perturbation gene effects were reported as Chronos score. Data were analyzed and plotted as box plot and floating bar plots using GraphPad Prism 8.0. Association between expression levels of Class I HDACs and overall survival of RMS patients was obtained using Williamson dataset (E-TABM-1202). Data has been downloaded at https://www.ebi.ac.uk/arrayexpress/experiments/E-TABM-1202/. Kaplan-Meier curve were generated using GraphPad Prism 9. The *p*-values are determinated by log rank test, where data was dichotomized into lowly expressed and highly expressed groups by percentiles. The best separation, smallest *p* value was then reported, accompanied by a Kaplan Meier picture. Correlations between HDAC3 expression and drug sensitivity in 9 RMS cell lines were retrieved from DepMap using the 22Q2 public dataset for gene expression and Drug Sensitivity AUC (CTD ^2) dataset for drug response data.

### Cell lines

RD (FN-RMS), RH4 and RH30 (FP-RMS) cell lines were purchased from ATCC (Rockville, MD, USA). RD18 (FN-RMS) were kindly provided from C. Ponzetto (Department of Oncology, University of Turin, Turin, Italy). RH2, JR1, and RH36 (FN-RMS) cell lines were provided by P. Houghton (Rockville, MD, United States). RH4 Cas9 and RH30 Cas9 were generously provided by C. R. Vakoc. All RMS cells were cultured in DMEM high-glucose (Invitrogen, Carlsbad, CA, United States) supplemented with 10% fetal bovine serum (FBS), 1% of an L-glutamine solution and 1% of a penicillin-streptomycin solution. HSMM (CC-2580) were purchased from Lonza (Walkersville, MD, USA). Vendors also supplied growth media with supplements and serum (CC-3245). MRC-5 human fibroblasts (CCL-171) were purchased from ATCC (Rockville, MD, USA) and cultured in Eagle’s Minimum Essential Medium, supplemented with 10% fetal bovine serum. Cells were cultured at 37 °C in a humidified atmosphere of 5% CO_2_/95% air and regularly checked for mycoplasma contamination. HSMMs were cultured in according to manufacturer’s instructions.

### Radiation exposure in vitro

Cells were irradiated at room temperature employing an x-6 MV photon linear accelerator, as previously described [19]. The flasks were irradiated with a total dose of 4 and 6 Gy at a rate of 2 Gy/min, maintaining a source-to-surface distance (SSD) of 100 cm. A slab of Perspex with a thickness of 1.2 cm was situated beneath the cell culture flasks to offset the build-up effect. The gantry angle was set at 180 degrees for irradiating the tumor cells. Non-irradiated control samples were treated in the same manner as the irradiated cells, except for the exposure to radiation. The absorbed dosage was measured using a Duplex dosimeter (PTW).

### RNA interference

RNA interference was performed with 100 nM final concentration siRNAs against human HDAC1 (GCCGGUCAUGUCCAAAGUAAU), HDAC2 (GUUGCUCGAUGUUGGACAUAU), HDAC3 (CCUGCAUUACGGUCUCUAUAA), HDAC8 (UUACGAUUGCGACGGAAAUUU) or with a non-targeting siRNA as control (SIC001) (Sigma-Aldrich, St Louis, MO, USA) using Oligofectamine (Invitrogen, Carlsbad, CA). The medium was replaced with fresh complete growth medium after 24 h of transfection.

### Lentiviral particle production

RH4 Cas9 and RH30 Cas9 cells were infected with lentiviral pLENTI-L3US2-RFPv3 expressing either a dual sgRNA sequences targeting HDAC3 or a non-targeting sgRNA sequence as reported in [29]. 2.6 μg of each sgRNA was transfected in HEK293T cells using Fugene 6 (Promega, Madison, WI, USA) with lentiviral packaging mix (SHP002, Sigma Aldrich, St Louis, MO, USA) in complete medium to produce lentiviral particles. 24 h later transfection the medium was replaced and 48 h later the lentiviral medium was collected. RH4 Cas9 and RH30 Cas9 were infected for 24 h in the presence of polybrene (8 μg/ml; Sigma-Aldrich, St Louis, MO, USA) and 10% FBS. Cells were harvested at 72 h post infection and plated for subsequent experiments.

### Biochemical HDAC1, -3, -4, -6, -8 evaluation

The HDAC3 inhibitor MC4448 was the A. Mai lab. MC4448 was tested against human recombinant HDAC1, − 3, − 4, − 6, and – 8 in 10-dose mode with 3-fold serial dilution starting from 200 μM solution (Fig. 5A). Inhibition values for each tested HDAC isozyme were measured with the homogeneous fluorescence release HDAC assay. Purified recombinant enzymes were incubated with serial diluted inhibitor at the indicated concentration. The deacetylase activities of HDACs 1, -3, -4, -6, and –8 in the presence of serial dilution of MC4448 were determined by assaying enzyme activity using the p53 residues 379–382 (Arg-His-Lys-Lys(Ac)AMC) to detect inhibitory activity against HDAC1, −3, −6, the diacetylated peptide from p53 residues 379–382 (Arg-His-Lys(Ac)-Lys(Ac)AMC) to detect inhibition against HDAC8, and the fluorogenic class Iia (Boc-Lys(trifluoroacetyl)-AMC) substrate to detect inhibition against HDAC4 [47]. Deacetylated/detrifluoroacetylated AMC-substrates were sensitive toward lysine peptidase, and free fluorogenic 4-methylcoumarin-7-amide was generated, which can be excited at 355 nm and observed at 460 nm (Reaction Biology Corporation, MD, USA). Data were analyzed on a plate-to-plate basis in relationship to the control and imported into analytical software (GraphPad Prism, CA, USA).

### Cell proliferation assay and drug treatment

1.5 × 10^3^ RH30 and RH4 cells were seeded on 384-well plates in complete growth medium and were treated with decreasing doses of MC4448 (10 µM-0.06 nM) or with DMSO after 24 h. Cell confluences were recorded by Celigo Image Cytometer (Nexcelom Bioscience, Lawrence, MA, USA) and IC_50_ values were calculated 72 h post treatment by using the GraphPad™ Prism version 8. For proliferation experiments, 1.5 × 10^3^ RH30 and RH4 cells were seeded on 384-well plates and, after 24 h (t0), media containing DMSO or MC4448 at the selected concentrations were added. Cell confluence was quantified under phase contrast every 24 h, until 72 h post-treatment using Celigo Image Cytometer (Nexcelom Bioscience, Lawrence, MA, USA). For all experiments RH30 and RH4 cells were treated with the IC_50_ values of MC4448, reported in the graphs, for 24 h. Treatment with 10 μM MEK/ERK inhibitor U0126, 5 μM of p38 inhibitor SB203580, or 10 μM JNKs inhibitor SP600125 (Sigma-Aldrich) was done for the times shown in the figures and started before radiation lasting for 24 h.

### Colony formation assay

Four × 10^3^ RD, RH4, RH30, RH4 Cas9, and RH30 Cas9 cells were seeded in 6-well plates in complete growth medium. Medium was refreshed once a week, and after two weeks cells were fixed and stained with a solution of 20% methanol/0.25% crystal violet. Images were acquired by iBright™ CL1500 Imaging System (Thermo Fisher Scientific, Lafayette, CO, USA). The number of colonies were counted. Plating Efficiency (PE), obtained by the ratio number of colonies/number of cells seeded, was calculated as reported in [28]. Triplicate assays were carried out in three independent experiments.

### Protein extraction and western blot

The whole-cell lysates were obtained by homogenizing cells in RIPA lysis buffer as previously described [94]. Detection was performed by Pierce™ ECL Western Blotting Substrate (Thermo Scientific™) or Western Lightning ECL Pro (PerkinElmer, Waltham, MA, USA). Filters were acquired by iBright™ CL1500 Imaging System (Thermo Fisher Scientific, Lafayette, CO, USA). Antibodies against HDAC1 (GTX100513), HDAC2 (GTX109642), HDAC3 (GTX109679-S), HDAC8 (GTX105074) were obtained from ProdottiGianni; Phospho-ATM (Ser 1981) (sc-47739), ATM (sc-377293), N-MYC (sc-53993), DNA-PKcs (sc-390849), p27 (sc-1641), and KU70 (sc-5309) were from Santa Cruz Biotechnology Inc., (Santa Cruz, CA, United States); Vinculin (V9131) was from Sigma (St Louis, MO, United States). Antibodies against Phospho-Histone H2A.X (Ser139) (9718), Histone H2AX (2595), Histone H3 (4499), p21 (2947), pospho-DNA-PKcs (68716), FOXO1 (28805), PARP (83732), Phospho-MEK (41G9), MEK (9122), Phospho-ERK (91015), ERK (9102), Phospho-p38 (9221), p38 (9212), Phospho-JNK (4668), JNK (9252), Phospho-cJun (2361), cJun (9165), RAD51(8875), PERK (D11A8), CHOP (L63F7), IRE1α (14C10), Ero1α (3264), Calnexin (C5C9), BIP (C50B12), ATF6 (D4Z8V), LC3A/B (D3U4C), SQSTM1/p62 (5114) and all secondary antibodies were obtained from Cell Signaling (Beverly, MA, USA); α-Tubulin (NB100-92249) and Histone acetyl-H3 (Lys9, Lys14) (NB21-1081) were from Novus Biologicals (Littleton, CO, United States). Mouse and rabbit secondary antibodies were obtained from Cell Signaling Technology, Inc., (Danvers, MA, USA). All antibodies were used in accordance with the manufacturer’s instructions.

### Annexin V determination

1.5 × 10^6^ cells were seeded into T75 flasks and after 24 h post IR they were harvested and cells suspension were incubated with FITC-conjugated Annexin V and 7-Aminoactinomycin D (7-AAD) in binding buffer for 15 min in the dark, using Annexin V apoptosis detection kit (BD Pharmingen, San Diego, CA, USA), according to manufacturer’s recommendations. Cells were nalysed using FACS CantoII equipped with a FACSDiva 6.1 CellQuest software (Becton Dickinson Instrument, San Josè, CA, USA).

### Caspase activity assay

Cells were seeded into 96-well black, flat bottom plates at a density of 5000 cells per well and incubated for 24 h to allow cell surface adhesion. The activity of Caspase-3/7 was determined after 24 h post IR with a Caspase-Glo-3/7 assay (Promega Company, Madison, WI, USA) according to the manufacturer’s protocol, using the EnSpire^®^ Multimode Plate Reader (PerkinElmer, Waltham, MA, USA).

### Cell cycle

RMS cells were harvested by trypsinization 24 h post IR, washed in cold phosphate buffered saline (PBS), and fixed in a cold solution of 50% PBS-5% FBS and 50% acetone/methanol (1:4 v/v) over night at 4 °C. Fixed cells were centrifuged for 5 min at 1200 rpm and the pellet was stained with 50 μg/ml propidium iodide (PI) (ThermoFisher Scientific, Rockford, USA) and 50 μg/ml Rnase (Sigma-Aldrich, St Louis, MO, USA) for 30 min in the dark at room temperature. FACSCantoII equipped with a FACSDiva 6.1 CellQuestTM software (Becton Dickinson Instrument, San Josè, CA, USA) was used for the acquisition and analysis of cell cycle.

### Cancer stem cells formation assay

10 × 10^3^ RH4, RH30, RH4 Cas9 and RH30 Cas9 cells were resuspended in 2 mL of Neuro Basal Medium, supplemented with 2X B27 (Thermo Fisher Scientific, Lafayette, CO, USA), 1% penicillin-streptomycin, 20 ng/mL EGF (130-097-749) and 20 ng/mL bFGF (130-093-840) both from Miltenyi (Miltenyi Biotec, Bergisch Gladbach, Germany) and plated in a low attachment 6-well plate (3471, Corning, New York, NY, USA). After ten days, images were captured by Leica microscope (Leica Microsystems, Mannhein, Germany) and the number of tumorspheres was counted.

### RNA isolation, cDNA synthesis and Real-Time qPCR

Total RNA was extracted using miRNeasy Micro Kit (Qiagen) according to the manufacturer’s protocol. RNA concentration was quantified using a NanoDrop^®^ 1000 Spectrophotometer (ThermoFisher Scientific). Reverse transcription was performed using the Improm-II Reverse Transcription System (Promega, Madison, WI, USA). The expression levels were measured by qRT-PCR for the relative quantification of the gene expression as described [95]. TaqMan gene assay (Applied Biosystems, Life Technologies, Carlsbad, CA, USA) for ATM (Hs00175892_m1), ATR (Hs00992123_m1), and Rad51 (Hs00947967_m1) were used. Values were normalized according to the glyceraldehyde-3-phosphate dehydrogenase (GAPDH) mRNA (Hs99999905_m1) levels. The QuantStudio 3 Real-Time PCR System (Applied Biosystems) was used for the measurements. The expression fold change was calculated by the 2-ΔΔCt method.

### Immunofluorescence and confocal microscopy

RH30 and RH4 cells were fixed 6 h post IR in 4% paraformaldehyde (PFA)/PBS for 10 min at room temperature, permeabilized in 0.5% Triton X-100/PBS for 15 min and incubated with rabbit phospho-H2AX (Ser139) (9718, Cell Signaling, Beverly, MA, USA) in 1% BSA/PBS. Alexa-488 goat α-rabbit (Invitrogen, Carlsbad, CA, USA) was used as secondary antibody. Cells were counterstained with DAPI and imaged by using the Olympus FV3000laser-scanning confocal microscope (Evident Scientific, Hamburg, Germany) equipped with 405 nm and 488 nm laser sources. Sequential confocal images were acquired using a UPLXAPO 60x oil immersion objective (1.42 numerical aperture) with a 1024 × 1024 format, scan speed 8 μs/pixel and z-step size of 0.3 μm. Maximal Intensity Projection (MIP) of each Z-reconstruction was obtained by FV31S-SW (version 2.4.1.198) Olympus software. Images were processed by Adobe Photoshop. The intensity average of phospho-H2AX fluorescence was calculated using a software from cytometric measurements in 8 digital images randomly selected and acquired for each cell sample using Olympus FV3000laser-scanning confocal microscope at 60x oil immersion objective (1.42 numerical aperture) with a 512 × 512 format and scan speed 8 μs/pixel and z-step size of 0.5 μm. Lasers; power, beam splitters, filter settings, pinhole diameter, and scan mode were the same for all examined samples of each staining. From 80 to 100 cells were counted for each sample analyzed.

### Neutral comet assay

DNA breakage induction was evaluated by Comet assay (single-cell gel electrophoresis) in non-denaturing conditions, as described [41]. Briefly, dust-free frosted-end microscope slides were dipped into molten agarose at 1% and left to dry. Cell pellets were resuspended in PBS and kept on ice to inhibit DNA repair. Cell suspensions were mixed with LMP agarose at 0.5% kept at 37 °C and pipetted onto agarose-covered surface of the slide. Agarose-embedded cells were lysed by submerging slides in lysis solution (30 mM EDTA, 0.1% sodium dodecyl sulphate (SDS)) and incubated at 4 °C, 45 min in the dark. After lysis, slides were washed in Tris Borate EDTA (TBE) 1X running buffer (Tris 90 mM; boric acid 90 mM; EDTA 4 mM) for 1 min. Electrophoresis was performed for 18 min in TBE 1X buffer at 1 V/cm. Slides were subsequently washed in distilled H_2_O and finally dehydrated in ice-cold methanol. Nuclei were stained with GelRed (1:1000) and visualized with a fluorescence microscope (Leica), using a 20X objective, connected to a CCD camera for image acquisition. At least 100 comets per cell line were analysed using CometAssay IV software (Perceptive instruments) and data from tail moments processed using Prism software.

### In vivo xenograft and radiation exposure

Female nude CD1 mice were provided by Charles River laboratories (Calco, Italy), aged 6–8 weeks and used for xenografts experiments. Animals were maintained in sterile conditions, with a 12 h light/12 h dark cycle, 18–23 °C ambient temperature and 40–60% humidity. RH30 Cas9 cells expressing either sgCTR or sgHDAC3, 5 × 10^6^ each, were subcutaneously injected in the left flank of the mice. When the masses were palpable, tumor sizes were measured, in a blinding manner, weekly throughout the treatment period and for the following two weeks by caliper. Experimental mice were randomized into the experimental groups following tumor inoculation and before irradiation treatments. For the in vivo IR model, mice were irradiated at room temperature using an Elekta 6-MV photon linear accelerator. Five fractions of 2 Gy were delivered every 2 days, for a total dose of 10 Gy. A dose rate of 1.5 Gy/min will be used with a SSD of 100 cm. Considering an α/β ratio of 3.2 Gy, this schedule guarantees a Biological Effective Dose (BED) to the tumor of 16.25 Gy, equal to 95% of the BED delivered by a single fraction of 6 Gy, i.e., 17.25 Gy [96–98]. Since the entire animals were irradiated, this schedule permitted the recovery of normal tissues. Prior to IR mice were anesthetized and were protected from off-target radiation by a 3 mm lead shield. Mouse experiments were conducted in compliance with the international, EU and national ethical requirements and were approved by the Italian Health Ministry 221/2022-PR (D9997.140).

### Statistical analysis

*P* values have been calculated with GraphPad Prism 8.0 using one-way analysis of variance ANOVA for multiple comparisons and Student’s T-test for single comparison. Statistical significance was set at *p* value less than 0.05. Significant *p* values were reported within the figures. We estimated the sample size considering no significant variation within each group of data. The principle of using the smallest sample size possible was adopted in planning the animal experiments. We estimated the sample size in order to detect a difference in averages of 2 standard deviations at the 0.05 level of significance with an 80% power. No sample-size calculation was performed for the in vitro experiments. Each condition was analyzed with three biological replicates, a standard for the experiments performed to account for reasonable range of variability among samples.

### Supplementary information

Supplementary Figures and figure legends

Uncropped Figures

## Data Availability

All the dataset analyzed in this work are publicly available. RNA-seq data from RMS patients in Fig. 1A are obtained from Gene Expression Omnibus under the accession code GSE108022. Correlation between patients’ survival and gene expression in Fig. 1B was obtained from R2 Genomics Analysis and Visualization Platform (https://hgserver1.amc.nl/cgi-bin/r2/main.cgi) using Williamson dataset (E-TABM-1202). Protein and mRNA expression data of RMS PDX in Figure S1A was retrieved from https://viz.stjude.cloud/st-jude-childrens-research-hospital/visualization/epigenetic-landscape-of-rhabdomyosarcoma-subtypes~67. Cell dependency data, cell lines expression data and Drug sensitivity data from CTD^2 dataset in Figure S1C-E and Figure S3A and B was downloaded from DepMap portal (https://depmap.org/portal/).
