# Supplementary material for: HDAC3 genetic and pharmacologic inhibition radiosensitizes fusion positive rhabdomyosarcoma by promoting DNA double-strand breaks
Source: Cell Death Discov. 2024 Aug 6;10:351. doi: 10.1038/s41420-024-02115-y (PMC11303816; doi:10.1038/s41420-024-02115-y)
Supplement: Supplementary file 1 — Supplementary Figures and figure legends [file 41420_2024_2115_MOESM1_ESM.pdf]

HDAC3 genetic and pharmacologic inhibition radiosensitizes fusion positive rhabdomyosarcoma by promoting DNA double-strand breaks

Matteo Cassandri[1,2,§], Antonella Porrazzo[1,2,§], Silvia Pomella[2,3,§], Beatrice Noce[4] , Clemens Zwergel[4], Francesca Antonella Aiello[2], Francesca Vulcano[5], Luisa Milazzo[5], Simona Camero[6], Deborah Pajalunga[5], Massimo Spada[7], Valeria Manzi[1,5], Giovanni Luca Gravina[8], Silvia Codenotti[9], Michela Piccione[10], Miriam Tomaciello[1], Michele Signore[11], Giovanni Barillari[3], Cinzia Marchese[12], Alessandro Fanzani[9], Biagio De Angelis[2], Concetta Quintarelli[2], Christopher R. Vakoc[13], Eleanor Y. Chen[14], Francesca Megiorni[6], Franco Locatelli[2,15], Sergio Valente[4], Antonello Mai[4,16], Rossella Rota[2,#], Francesco Marampon[1,#,*].

- **Supplementary Figures**
- **Supplementary figure legends**

Figure S1

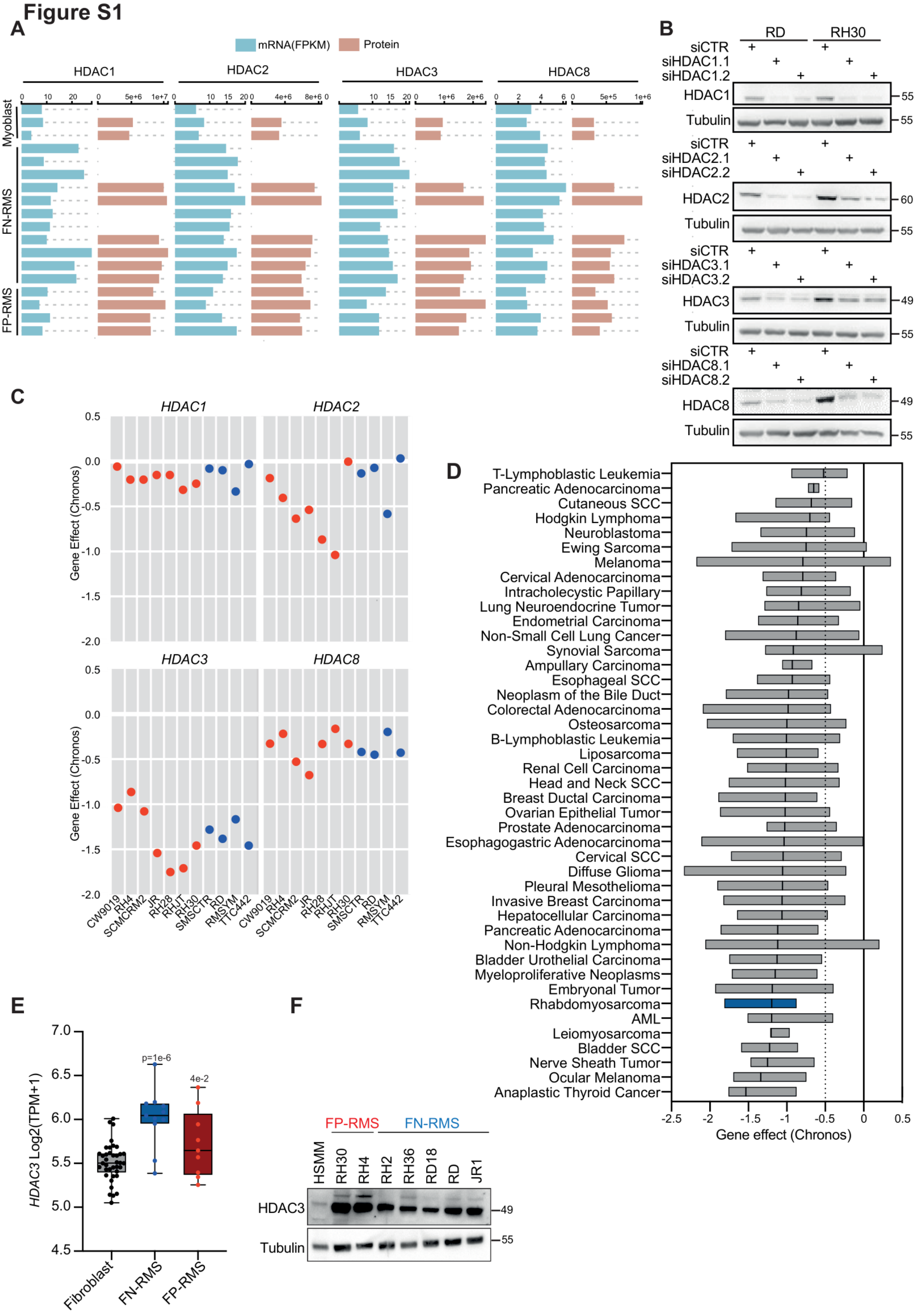
A
mRNA(FPKM)
Protein
HDAC1
HDAC2
HDAC3
HDAC8
Myoblast
FN-RMS
FP-RMS
B
RD
RH30
siCTR
siHDAC1.1
siHDAC1.2
HDAC1
Tubulin
siHDAC2.1
siHDAC2.2
HDAC2
siHDAC3.1
siHDAC3.2
HDAC3
siHDAC8.1
siHDAC8.2
HDAC8
55
60
49
C
Gene Effect (Chronos)
CW9019
RH4
SCMCRM2
JR
RH28
RHJT
RH30
SMSCTR
RD
RMSYM
TTC442
D
T-Lymphoblastic Leukemia
Pancreatic Adenocarcinoma
Cutaneous SCC
Hodgkin Lymphoma
Neuroblastoma
Ewing Sarcoma
Melanoma
Cervical Adenocarcinoma
Intracholecystic Papillary
Lung Neuroendocrine Tumor
Endometrial Carcinoma
Non-Small Cell Lung Cancer
Synovial Sarcoma
Ampullary Carcinoma
Esophageal SCC
Neoplasm of the Bile Duct
Colorectal Adenocarcinoma
Osteosarcoma
B-Lymphoblastic Leukemia
Liposarcoma
Renal Cell Carcinoma
Head and Neck SCC
Breast Ductal Carcinoma
Ovarian Epithelial Tumor
Prostate Adenocarcinoma
Esophagogastric Adenocarcinoma
Cervical SCC
Diffuse Glioma
Pleural Mesothelioma
Invasive Breast Carcinoma
Hepatocellular Carcinoma
Pancreatic Adenocarcinoma
Non-Hodgkin Lymphoma
Bladder Urothelial Carcinoma
Myeloproliferative Neoplasms
Embryonal Tumor
Rhabdomyosarcoma
AML
Leiomyosarcoma
Bladder SCC
Nerve Sheath Tumor
Ocular Melanoma
Anaplastic Thyroid Cancer
Gene effect (Chronos)
E
HDAC3 Log2(TPM+1)
p=1e-6
4e-2
Fibroblast
FN-RMS
FP-RMS
F
FP-RMS
FN-RMS
HSMM
RH30
RH4
RH2
RH36
RD18
RD
JR1
HDAC3
Tubulin
49
55

Figure S1. HDAC3 is highly expressed in RMS and correlates with drugs that induce DSBs. **A.** Expression of Class-I HDACs at mRNA (left) and protein (right) level on PDX in orthotopic RMS patient-derived xenografts (PDX) compared to normal myoblasts and myotubes. Data derive from https://pecan.stjude.cloud/proteinpaint/study/RHB2018. FPKM, Fragments per Kilobase Milion reads. **B.** Scatter plot for CRISPR Class-I HDACs depletion from Achilles project across FN-RMS and FP-RMS cell lines (https://depmap.org/portal/achilles/). **C.** Floating bars plot for CRISPR HDAC3 depletion from Achilles project across different tumor types (https://depmap.org/portal/achilles/). Data presented as the interval between minimum and maximum Chronos score value, black bar shows the median. Full line identifies a score of 0 (equivalent to a gene that is not essential in a given cell line) and dashed line identifies a score of -0.5 (equivalent to a gene that is dependent in a given cell line). **D.** Box plot for RNA-seq data from Achilles project depicting HDAC3 expression in FP-RMS and FN-RMS cell lines compared to normal human fibroblast. TPM, Transcripts per Million. **E.** Representative western blot (n = 3 independent experiments) of the indicated proteins in HSMM (normal control cells), FP-RMS and FN-RMS cell lines. α-TUBULIN is the loading control.

Figure S2

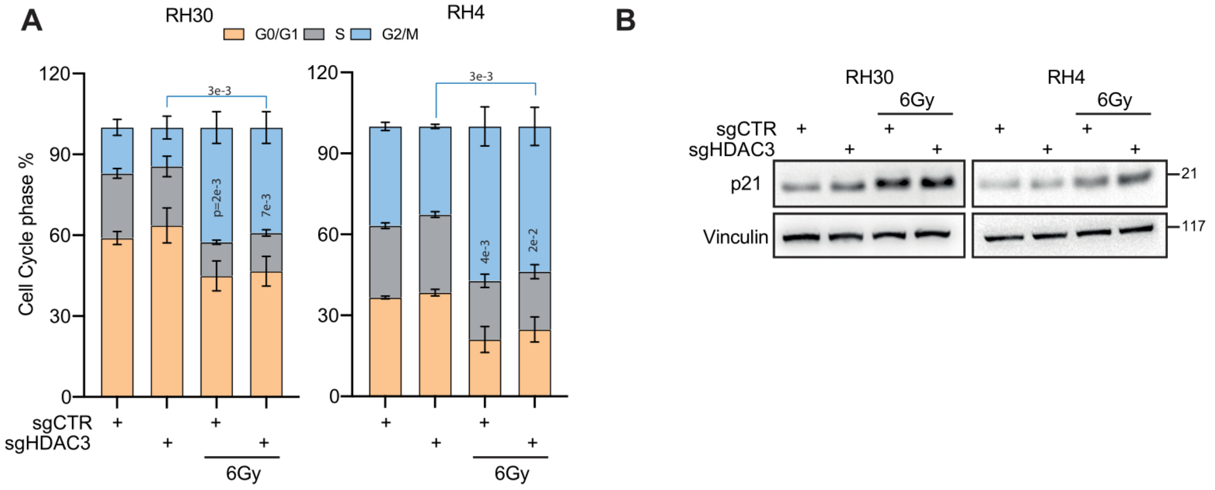

Figure S2. HDAC3 depletion in RH30 and RH4 cells does not influence the cell cycle after IR. A Histogram depicting the percentage of RH30 and RH4 HDAC3-depleted cells in G0/G1, S, and G2/M phases of cell cycle before and after 24 h post IR (6Gy). Graph represents the mean of three independent experiments ±SD, one-way ANOVA. **B** Representative western blot (n=3) of RH30 and RH4 cells infected with either sgControl (sgCTR) or sgRNA against HDAC3 (sgHDAC3) at 72 hours post-infection and then treated or not with 6Gy of IR. Vinculin was used as loading control.

Figure S3

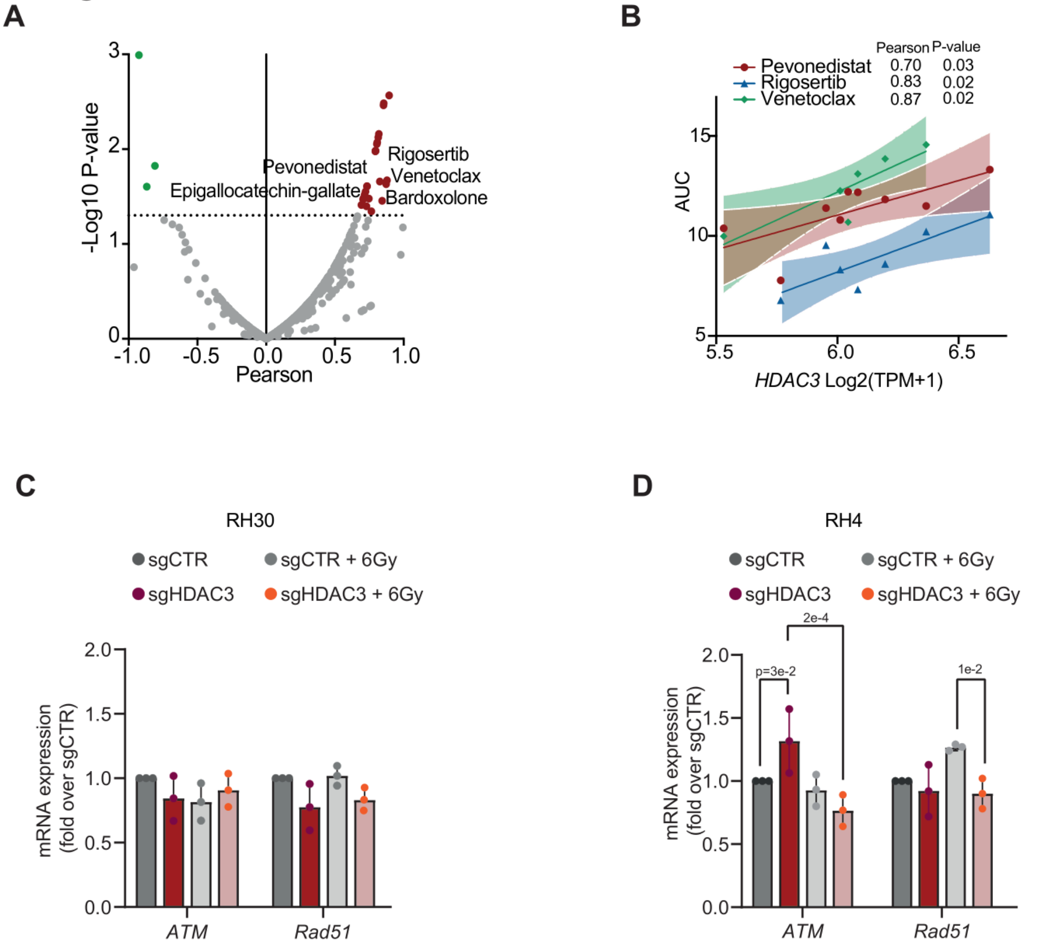

Figure S3. HDAC3 expression correlates with DNA damage inducing drugs sensitivity.

A. Volcano plot showing correlation between HDAC3 expression and drug response in 9 RMS cell lines from drug sensitivity AUC (CTD^2) (546 drugs). **B.** Pearson correlation analysis between HDAC3 expression and selected drug AUC. Low AUC indicates high drug efficacy while high AUC indicates low drug efficacy **C.** Histogram representing the fold-change expression of ATM, ATR, and Rad51 genes in sgHDAC3, sgCTR +IR, and sgHDAC3 +IR respect with to untreated sgCTR in RH30 cells. **D.** Histogram representing the fold-change expression of ATM, ATR, and Rad51 genes in sgHDAC3, sgCTR +IR, and sgHDAC3 +IR respect with to untreated sgCTR in RH4 cells. n = 3 independent experiments, data presented as mean values ± SD, one-way ANOVA.

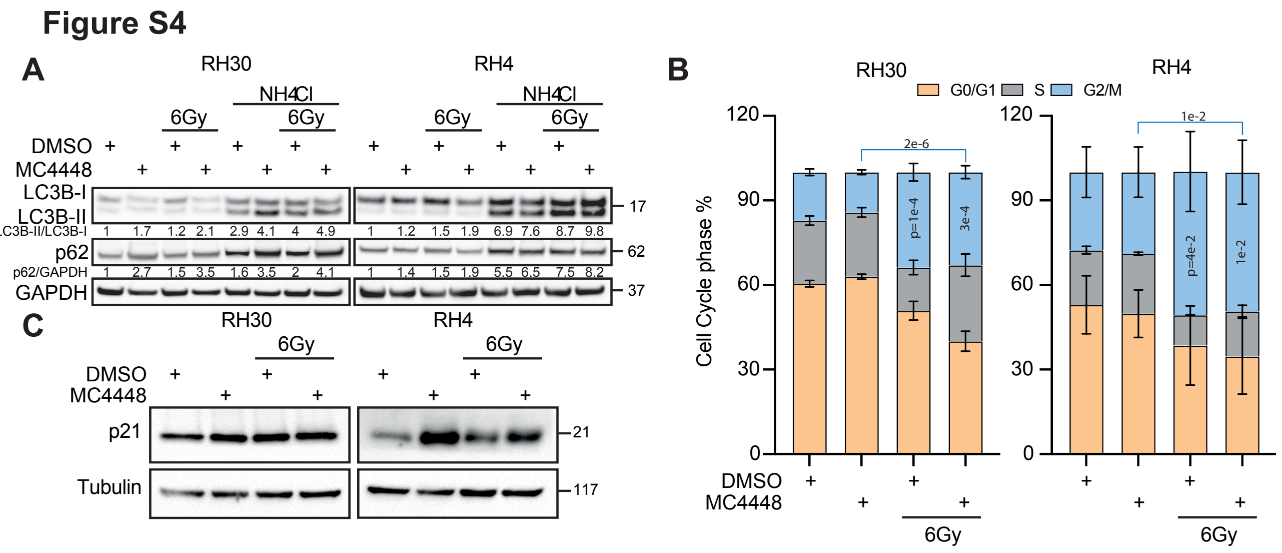

Figure S4. The combination of IR and MC4448 does not affect cell cycle in FP-RMS.

A. Representative western blot (n=3) depicting levels of LC3 and p62 of RH30 and RH4 cells treated with either DMSO or MC4448 IC50 for 24 hours, treated or not with 6Gy of IR and treated or not with NH_4Cl 25 µM to block autophagy. GAPDH was used as loading control. **B.** Histogram depicting the percentage of RH30 and RH4 cells treated with MC4448 in G0/G1, S, and G2/M phases of cell cycle before and after 24 h post IR (6Gy). Graph represents the mean of three independent experiments ±SD, one-way ANOVA. **C.** Representative western blot (n=3) of RH30 and RH4 cells treated MC4448 or DMSO after 24 hours post-IR with 6Gy of IR. Vinculin was used as loading control.

Figure S5

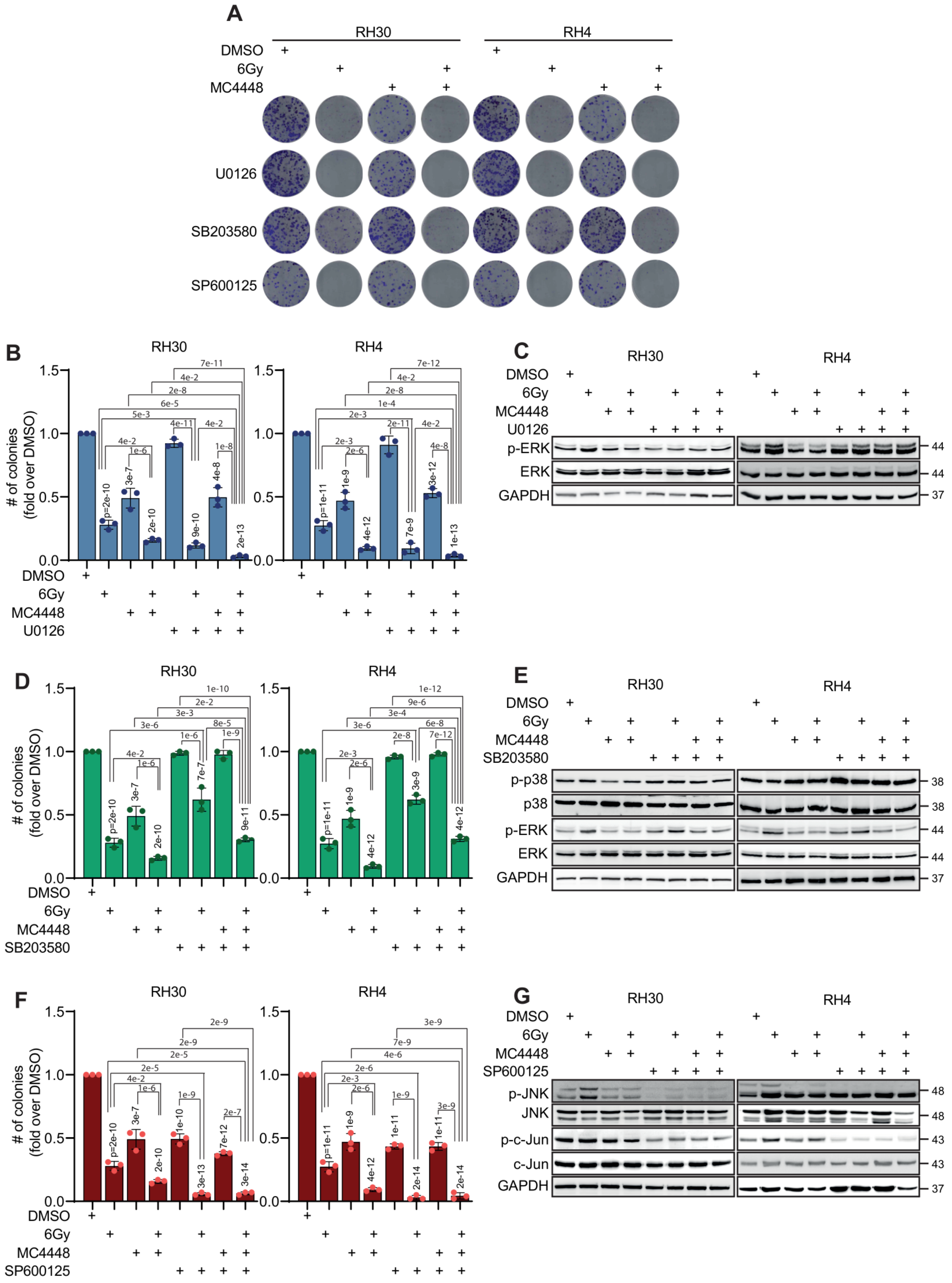
A
RH30
RH4
DMSO
6Gy
MC4448
U0126
SB203580
SP600125
B
C
D
E
F
G
# of colonies (fold over DMSO)
p-ERK
ERK
GAPDH
p-p38
p38
p-JNK
JNK
p-c-Jun
c-Jun

Figure S5. MEK/ERK inhibition enhances MC4448 radiosensitization effects.

A. Representative images of RH30 and RH4 colonies stained with crystal violet 12 days post seeding. FP-RMS cells were treated with either MC4448 IC50 (40 nM for RH30 and 58 nM for RH4), U0126 (10 μM), SB203580 (5 μM) and SP600125 (10 μM) or DMSO for 24 hours and then irradiated or not with 6 Gy. The cells were processed 6 hours post IR. **B.** Histograms depicting the plating efficiency of RH30 and RH4 treated with either MC4448, U0126 or DMSO for 24 hours and then irradiated or not with 6 Gy. Graph represents the mean of three independent experiments ±SD, one-way ANOVA. Exact p-values are reported in the figure. **C.** Representative western blot (n=3) depicting levels of pERK and ERK of RH30 and RH4 cells treated as in B. GAPDH was used as loading control. **D.** Histograms depicting the plating efficiency of RH30 and RH4 treated with either MC4448, SB203580 or DMSO for 24 hours and then irradiated or not with 6 Gy. Graph represents the mean of three independent experiments ±SD, one-way ANOVA. Exact p-values are reported in the figure. **E.** Representative western blot (n=3) depicting levels of p-p38, p38, p-ERK and ERK of RH30 and RH4 cells treated as in D. GAPDH was used as loading control. **F.** Histograms depicting the plating efficiency of RH30 and RH4 treated with either MC4448, SP600125 or DMSO for 24 hours and then irradiated or not with 6 Gy. Graph represents the mean of three independent experiments ±SD, one-way ANOVA. Exact p-values are reported in the figure. **G.** Representative western blot (n=3) depicting levels of p-JNK, JNK, p-c-JUN and c-JUN of RH30 and RH4 cells treated as in F. GAPDH was used as loading control.

Figure S6

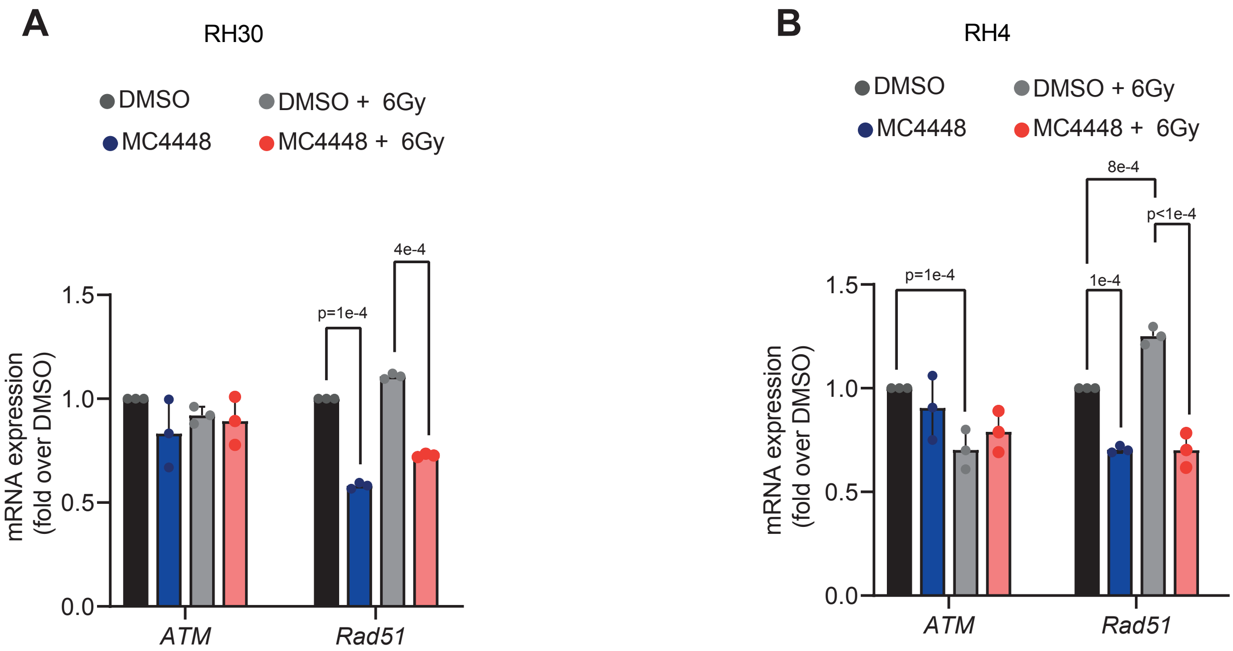

Figure S6. Fold-change expression of DDR genes after IR in RH30 and RH4 cells treated with MC4448.

A Histogram representing the fold-change expression of ATM, ATR, and Rad51 genes in RH30 cells treated with MC4448 or not after IR compared to untreated controls. **B** Histogram representing the fold-change expression of ATM, ATR, and Rad51 genes in RH4 cells treated with MC4448 or not after IR compared to untreated controls. n = 3 independent experiments, data presented as mean values ± SD, one-way ANOVA.
