# Supplementary material for: HDAC3 genetic and pharmacologic inhibition radiosensitizes fusion positive rhabdomyosarcoma by promoting DNA double-strand breaks
Source: Cell Death Discov. 2024 Aug 6;10:351. doi: 10.1038/s41420-024-02115-y (PMC11303816; doi:10.1038/s41420-024-02115-y)
Supplement: Supplementary file 2 — Uncropped Figures [file 41420_2024_2115_MOESM2_ESM.pdf]

Figure 1

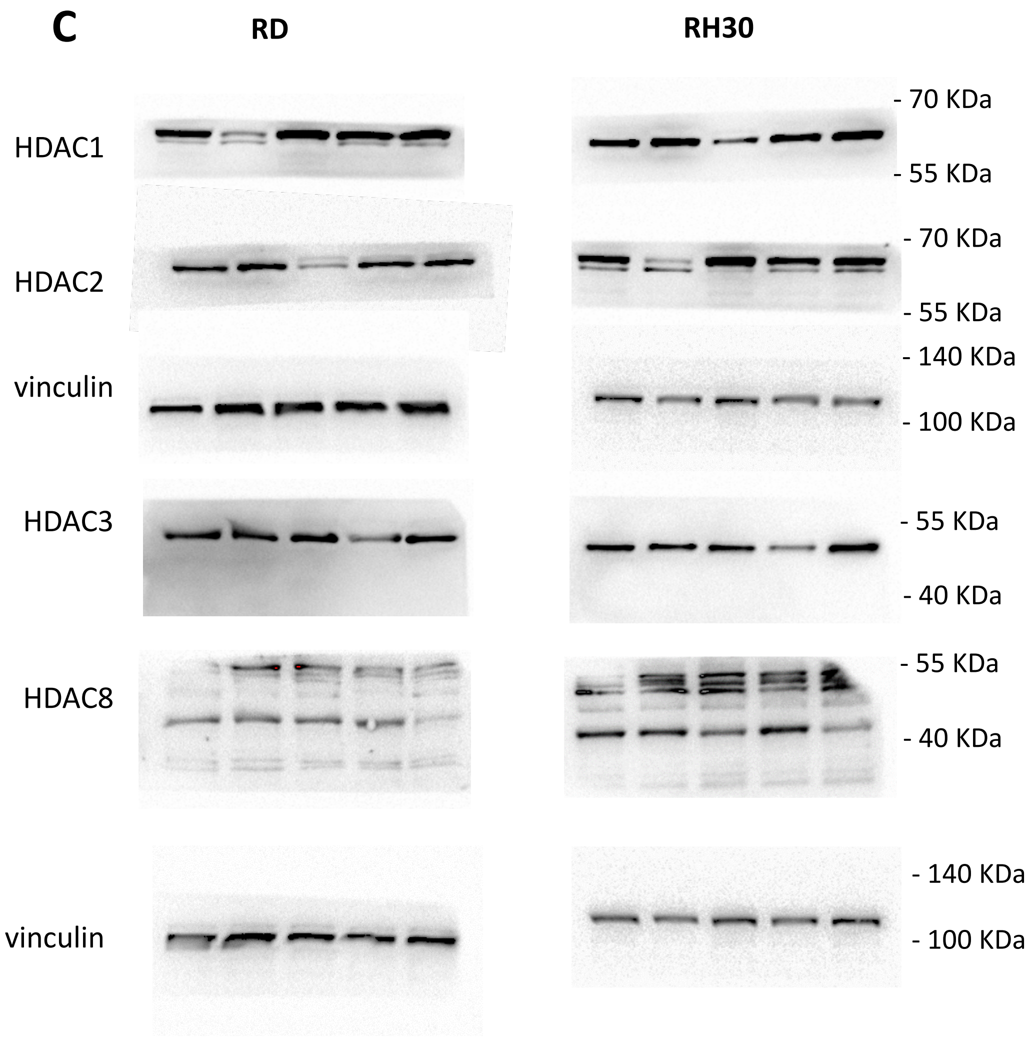
C
RD
RH30
HDAC1
HDAC2
vinculin
HDAC3
HDAC8
vinculin
- 70 KDa
- 55 KDa
- 70 KDa
- 55 KDa
- 140 KDa
- 100 KDa
- 55 KDa
- 40 KDa
- 55 KDa
- 40 KDa
- 140 KDa
- 100 KDa

Figure 2

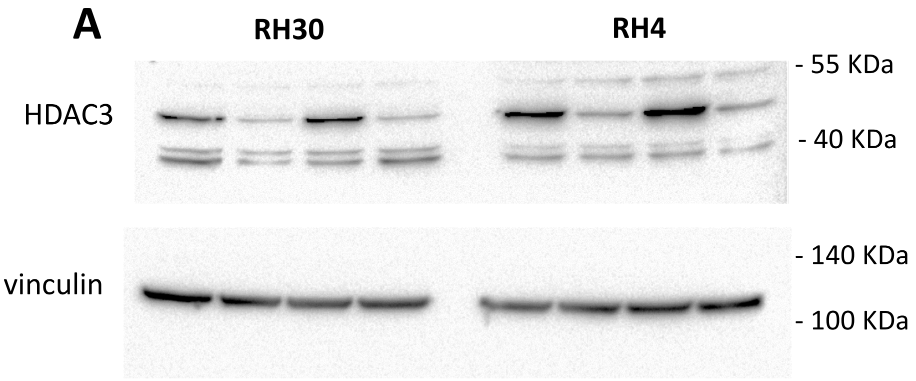

Figure 2

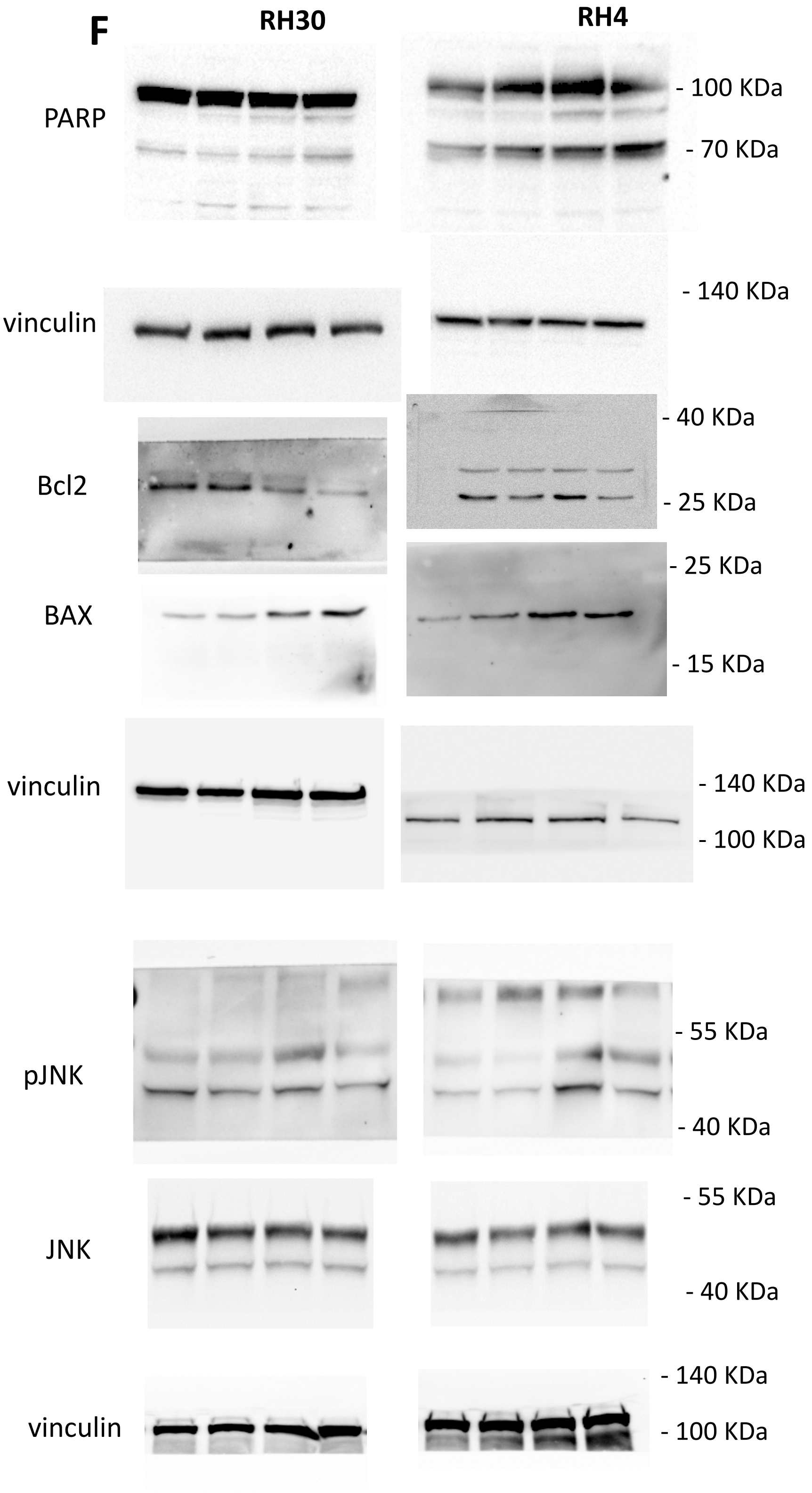
F
RH30
RH4
PARP
- 100 KDa
- 70 KDa
vinculin
- 140 KDa
Bcl2
- 40 KDa
- 25 KDa
BAX
- 25 KDa
- 15 KDa
vinculin
- 140 KDa
- 100 KDa
pJNK
- 55 KDa
- 40 KDa
JNK
- 55 KDa
- 40 KDa
vinculin
- 140 KDa
- 100 KDa

Figure 2

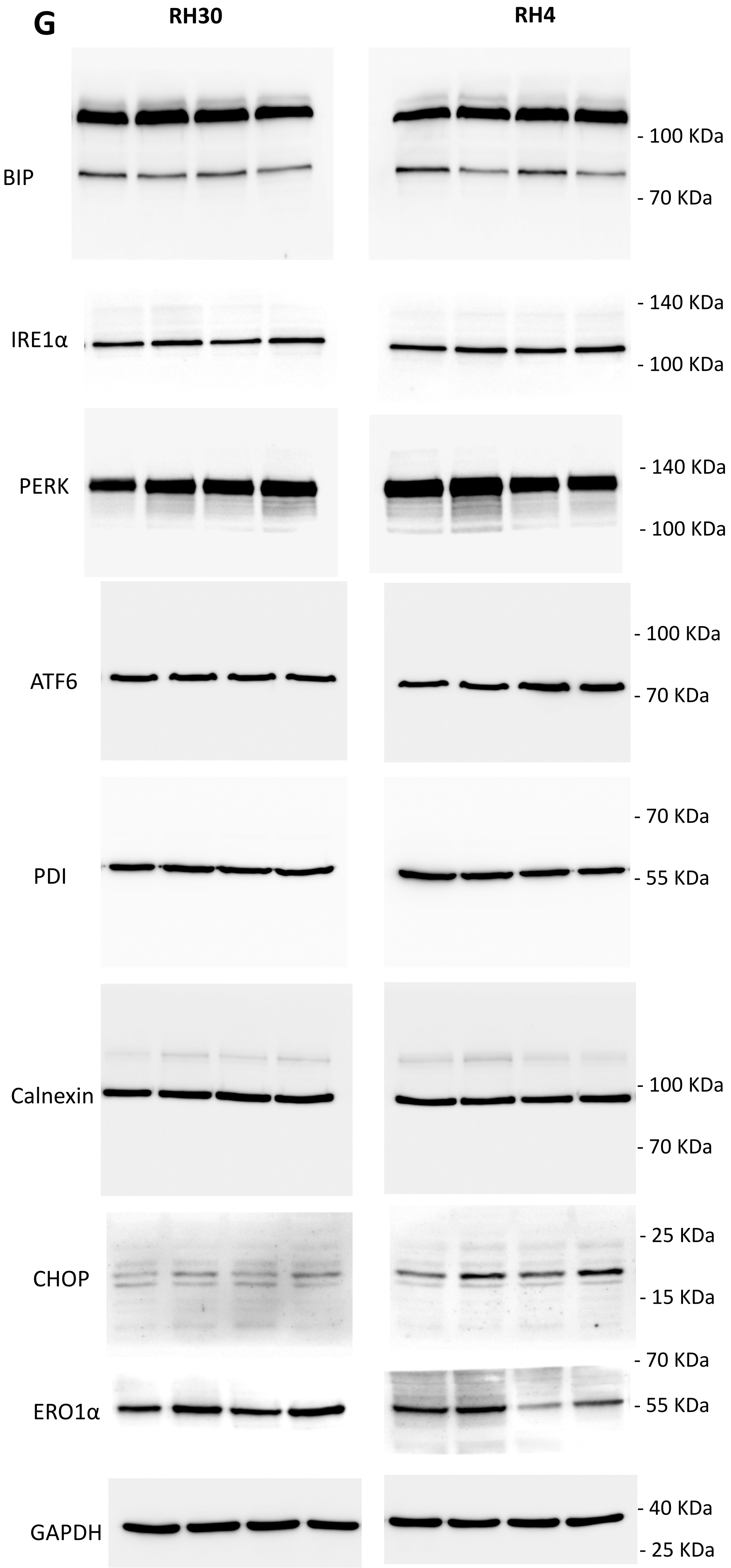

G
RH30
RH4
BIP
- 100 KDa
- 70 KDa
IRE1α
- 140 KDa
- 100 KDa
PERK
- 140 KDa
- 100 KDa
ATF6
- 100 KDa
- 70 KDa
PDI
- 70 KDa
- 55 KDa
Calnexin
- 100 KDa
- 70 KDa
CHOP
- 25 KDa
- 15 KDa
ERO1α
- 70 KDa
- 55 KDa
GAPDH
- 40 KDa
- 25 KDa

Figure 3

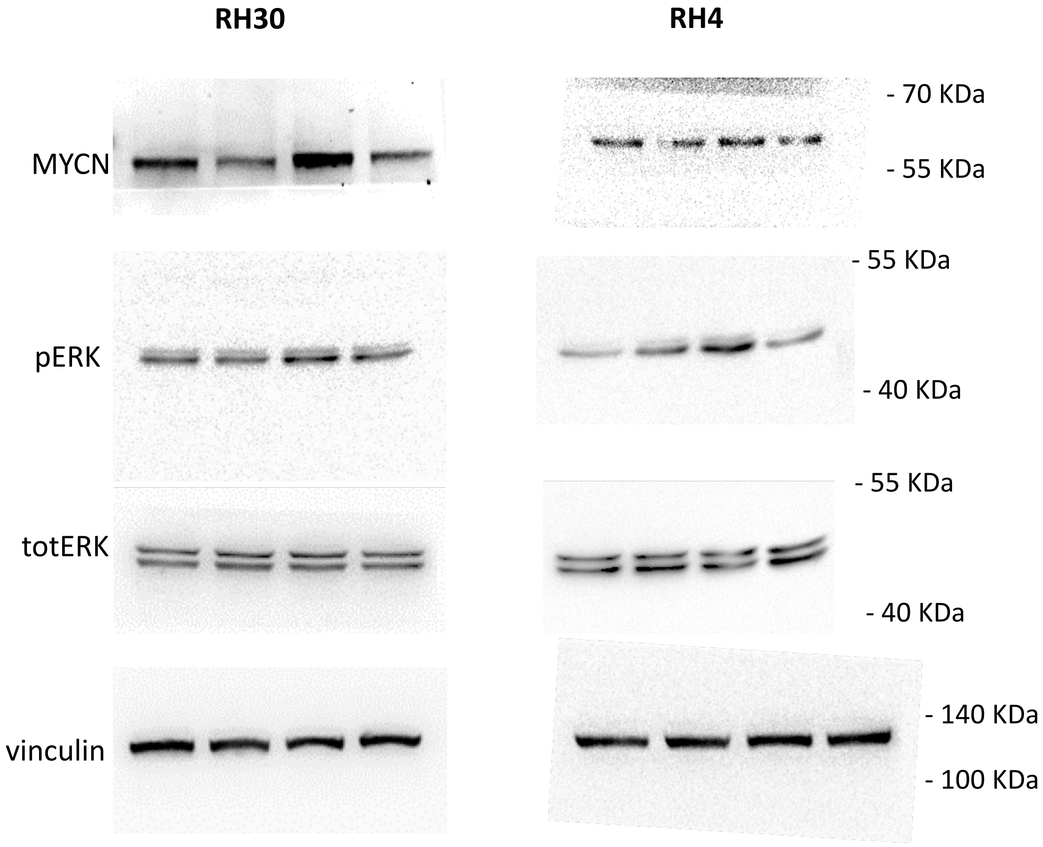
RH30
RH4
MYCN
pERK
totERK
vinculin
- 70 KDa
- 55 KDa
- 55 KDa
- 40 KDa
- 55 KDa
- 40 KDa
- 140 KDa
- 100 KDa

Figure 4

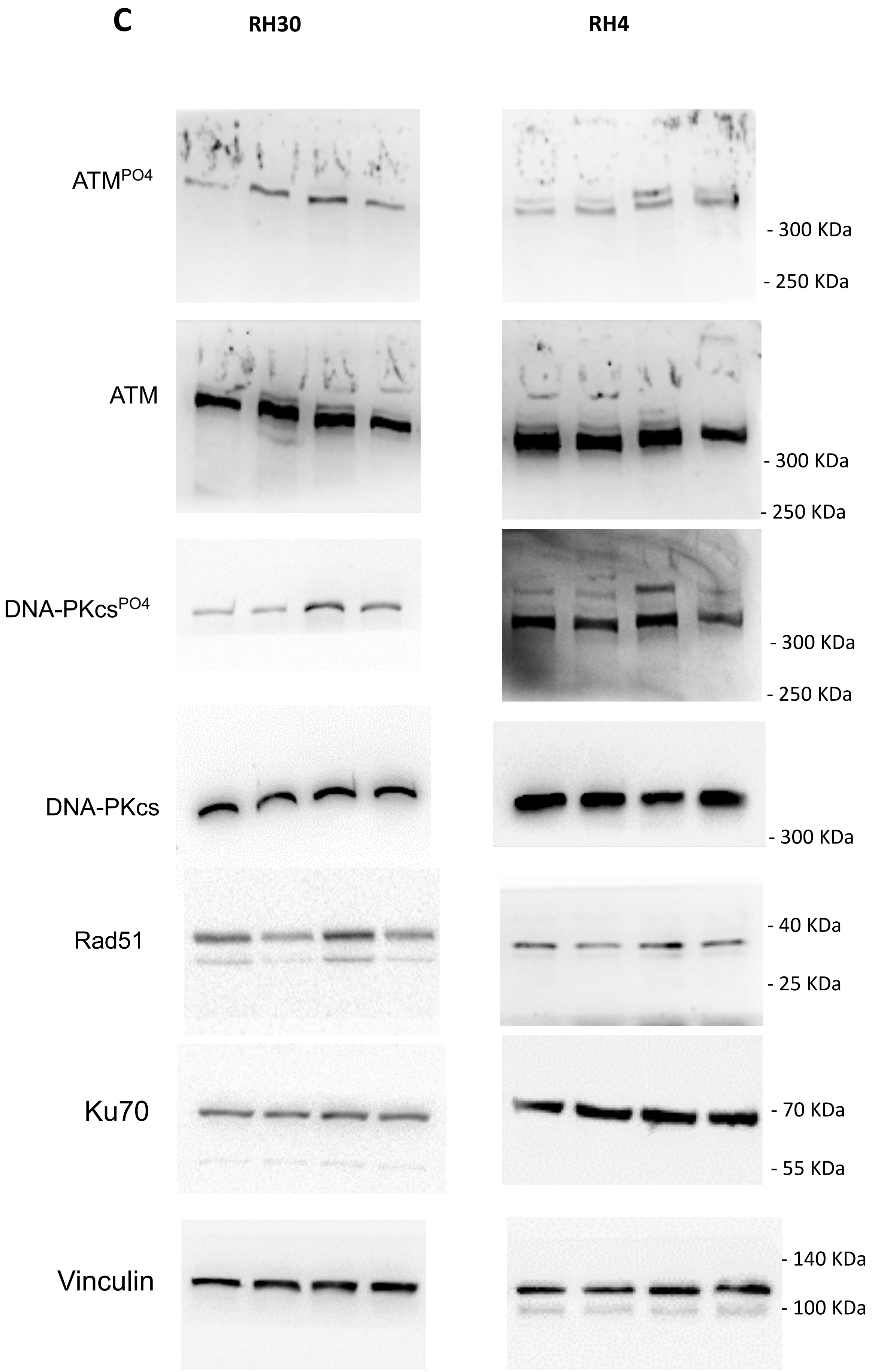
C
RH30
RH4
ATM^PO4
ATM
DNA-PKcs^PO4
DNA-PKcs
Rad51
Ku70
Vinculin
- 300 KDa
- 250 KDa
- 300 KDa
- 250 KDa
- 300 KDa
- 250 KDa
- 300 KDa
- 40 KDa
- 25 KDa
- 70 KDa
- 55 KDa
- 140 KDa
- 100 KDa

Figure 5

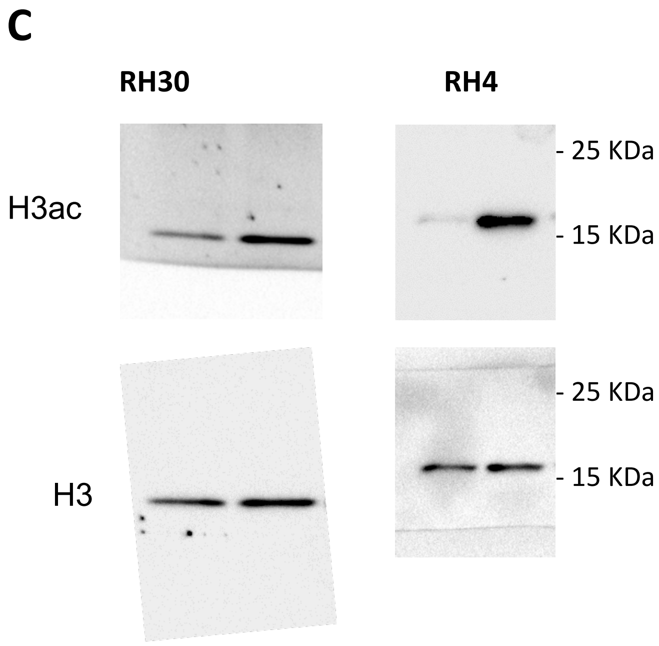

Figure 6

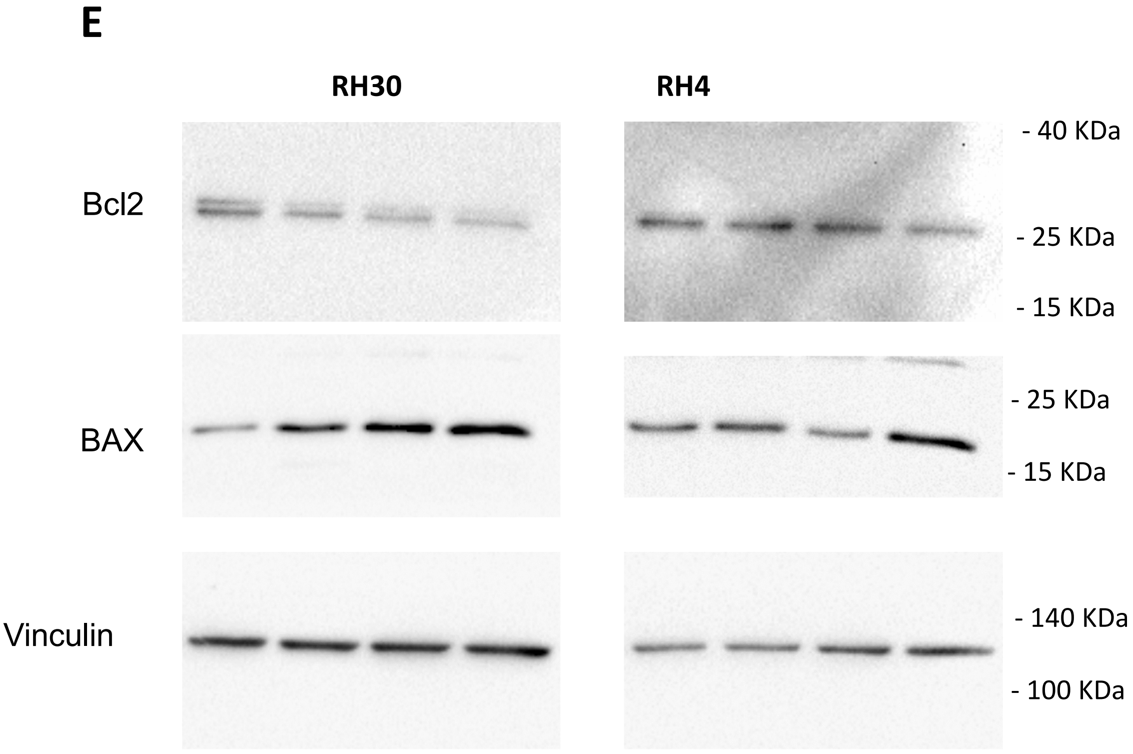
E
RH30
RH4
Bcl2
BAX
Vinculin
- 40 KDa
- 25 KDa
- 15 KDa
- 25 KDa
- 15 KDa
- 140 KDa
- 100 KDa

Figure 6

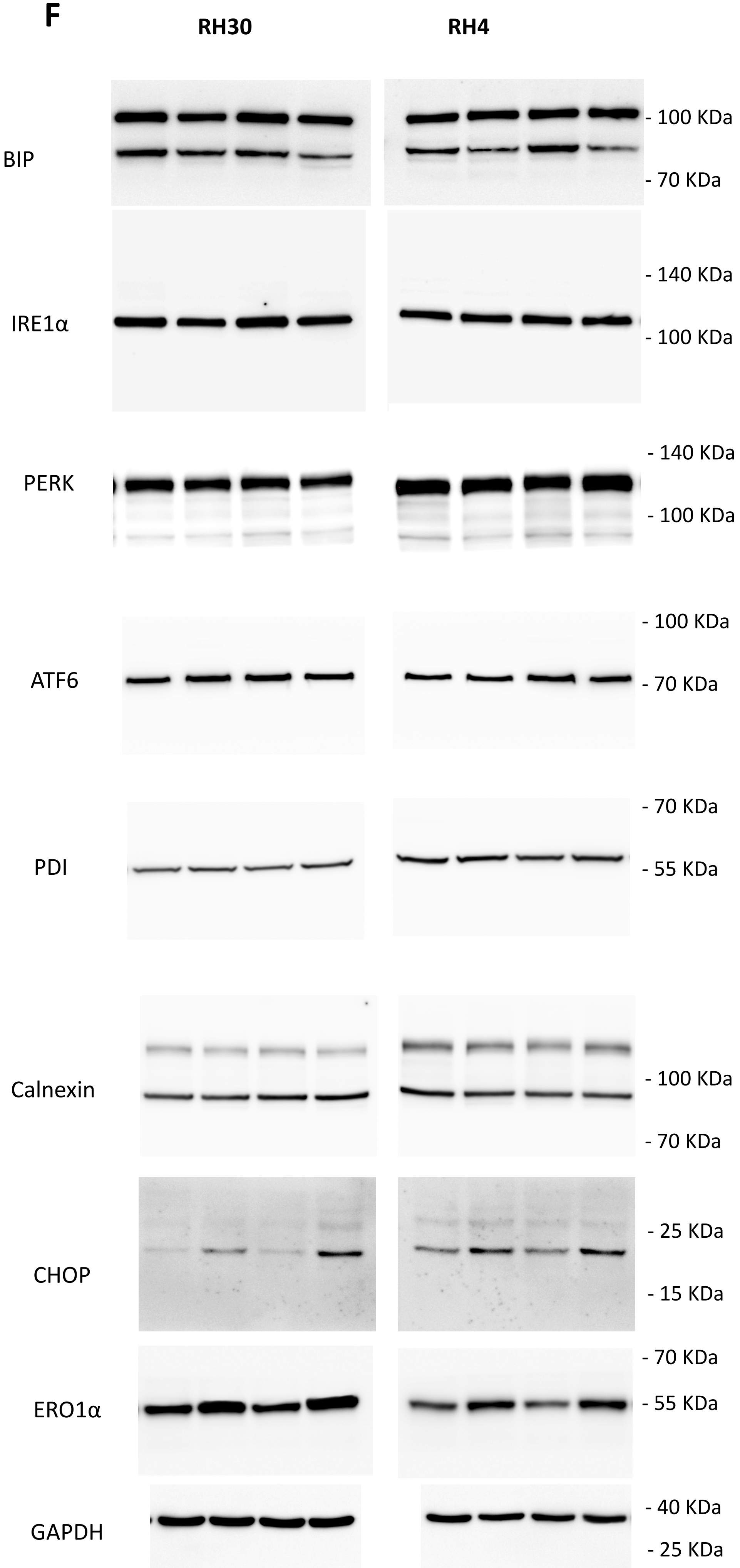
F
RH30
RH4
BIP
- 100 KDa
- 70 KDa
IRE1α
- 140 KDa
- 100 KDa
PERK
- 140 KDa
- 100 KDa
ATF6
- 100 KDa
- 70 KDa
PDI
- 70 KDa
- 55 KDa
Calnexin
- 100 KDa
- 70 KDa
CHOP
- 25 KDa
- 15 KDa
ERO1α
- 70 KDa
- 55 KDa
GAPDH
- 40 KDa
- 25 KDa

Figure 7

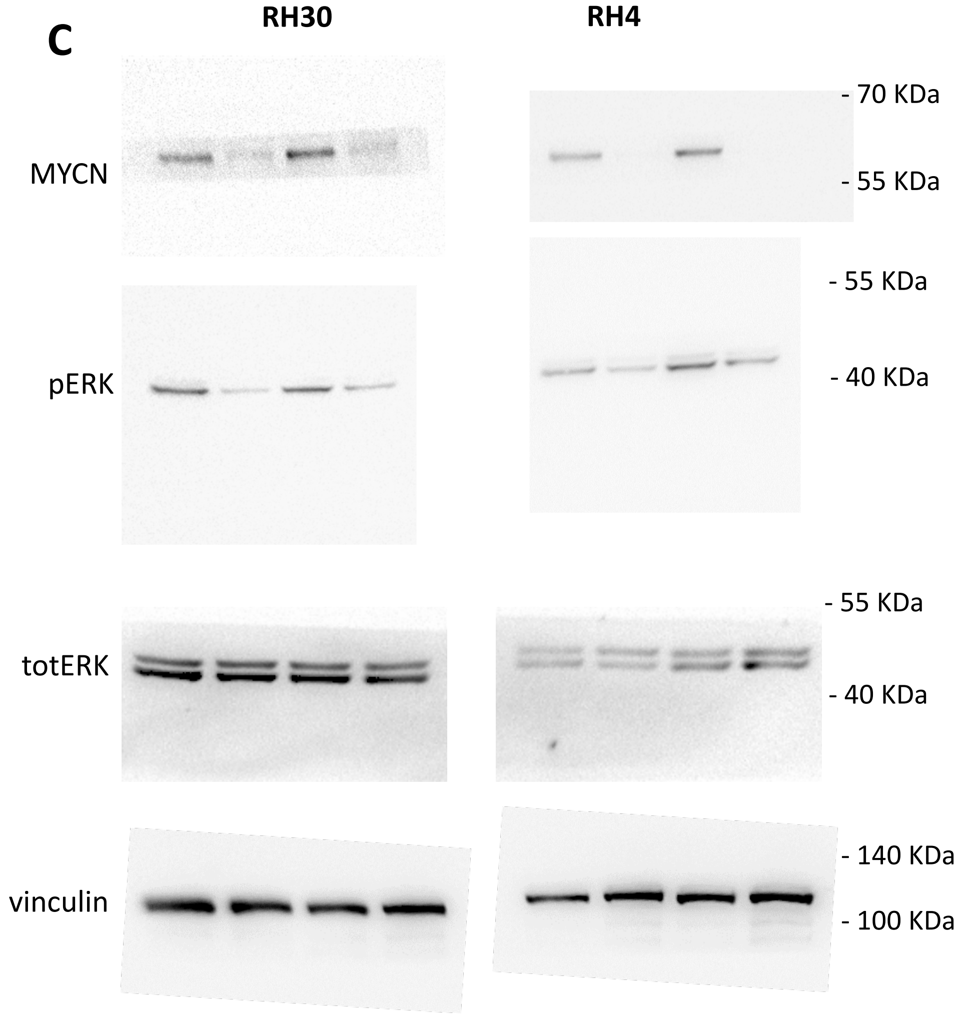
C
RH30
RH4
MYCN
pERK
totERK
vinculin
- 70 KDa
- 55 KDa
- 55 KDa
- 40 KDa
- 55 KDa
- 40 KDa
- 140 KDa
- 100 KDa

Figure 8

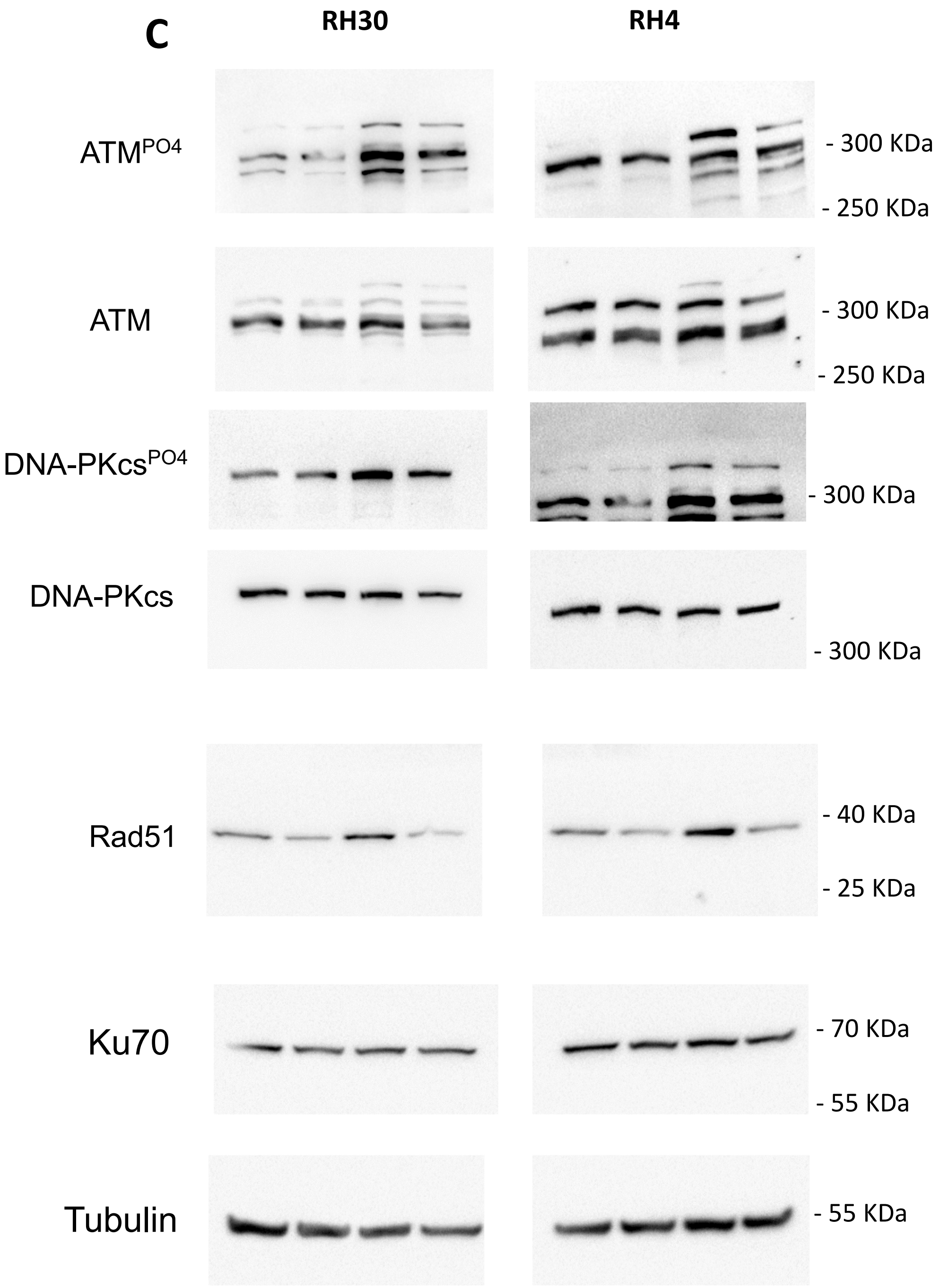
C
RH30
RH4
ATM^PO4
ATM
DNA-PKcs^PO4
DNA-PKcs
Rad51
Ku70
Tubulin
- 300 KDa
- 250 KDa
- 300 KDa
- 250 KDa
- 300 KDa
- 300 KDa
- 40 KDa
- 25 KDa
- 70 KDa
- 55 KDa
- 55 KDa

Figure S1

B

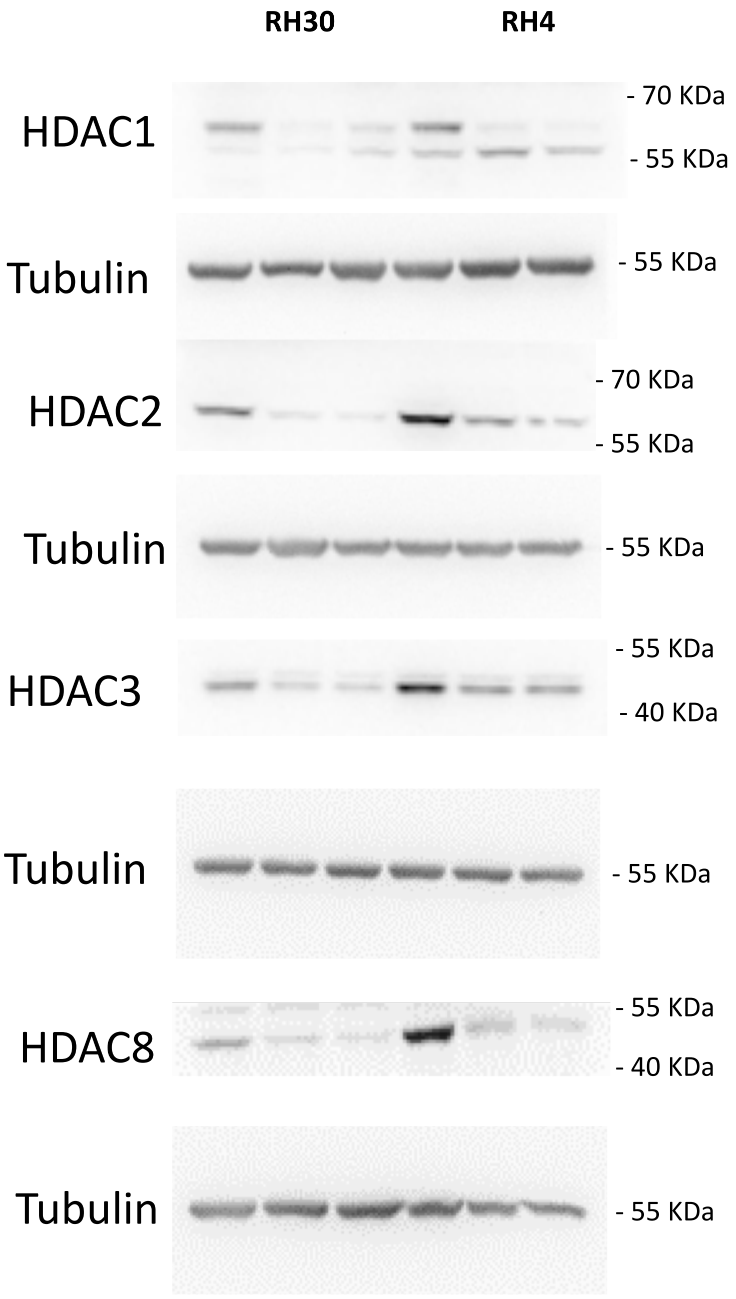

RH30
RH4
HDAC1
- 70 KDa
- 55 KDa
Tubulin
- 55 KDa
HDAC2
- 70 KDa
- 55 KDa
Tubulin
- 55 KDa
HDAC3
- 55 KDa
- 40 KDa
Tubulin
- 55 KDa
HDAC8
- 55 KDa
- 40 KDa
Tubulin
- 55 KDa

F

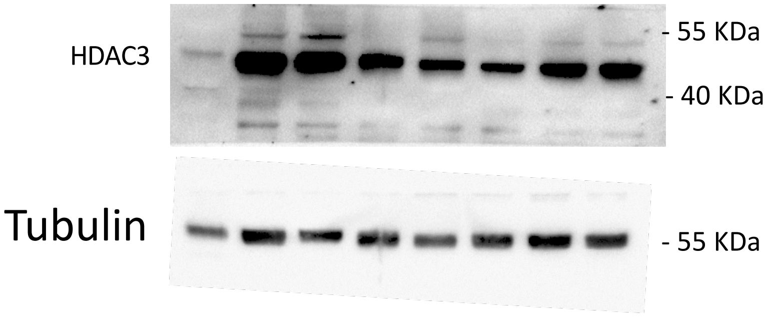

HDAC3
- 55 KDa
- 40 KDa
Tubulin
- 55 KDa

Figure S2

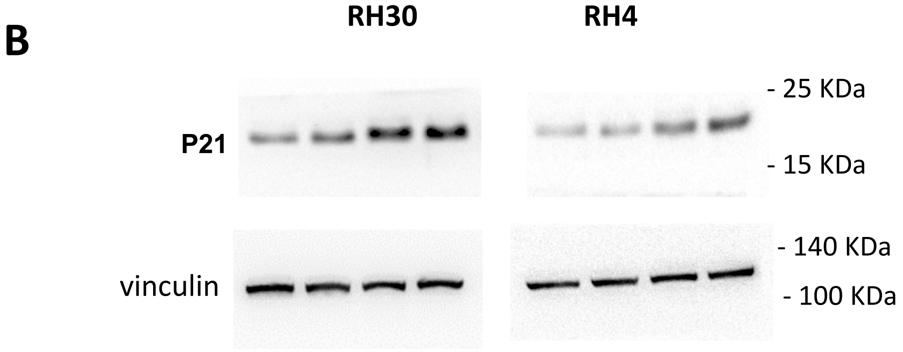

Figure S4

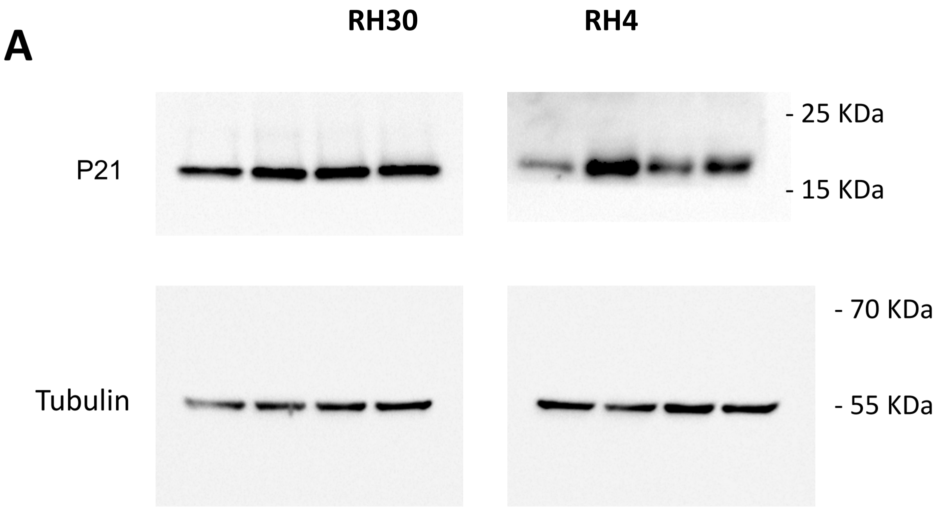
A
RH30
RH4
P21
Tubulin
- 25 KDa
- 15 KDa
- 70 KDa
- 55 KDa

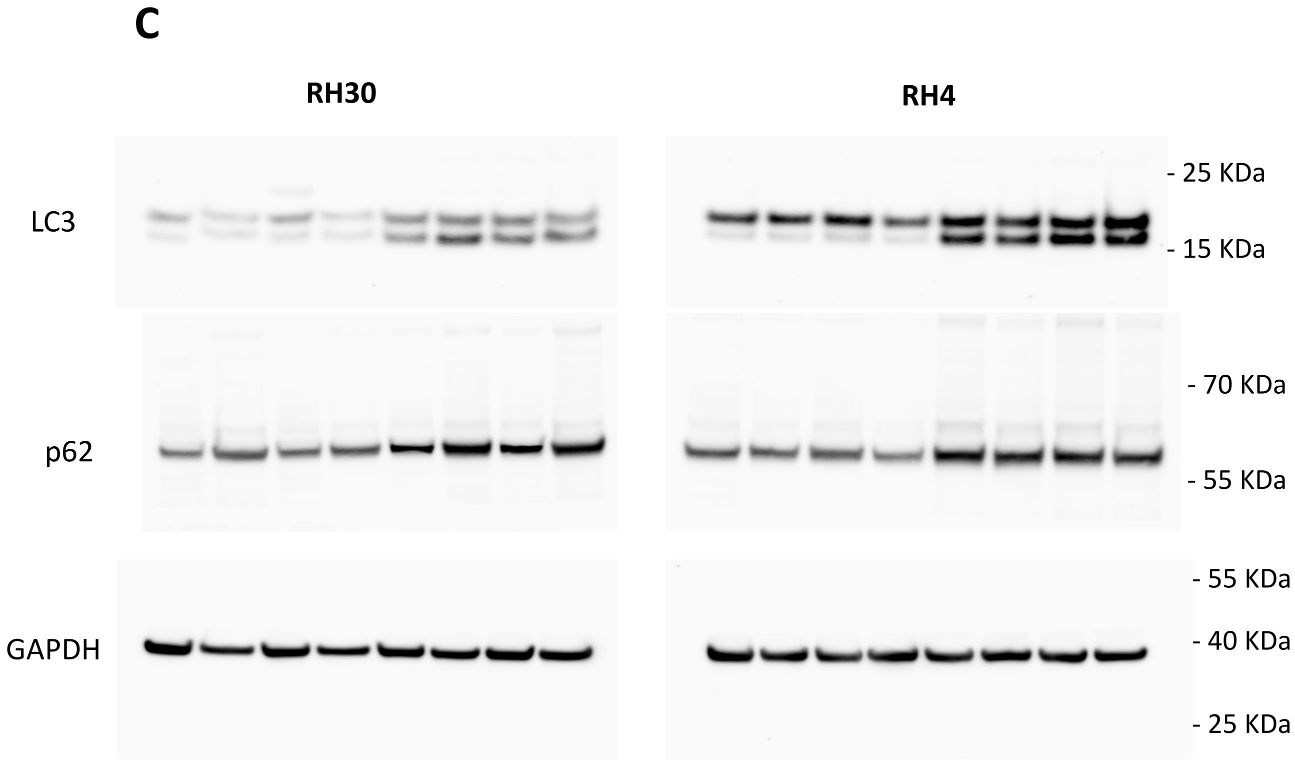
C
RH30
RH4
LC3
p62
GAPDH
- 25 KDa
- 15 KDa
- 70 KDa
- 55 KDa
- 55 KDa
- 40 KDa
- 25 KDa

Figure S4

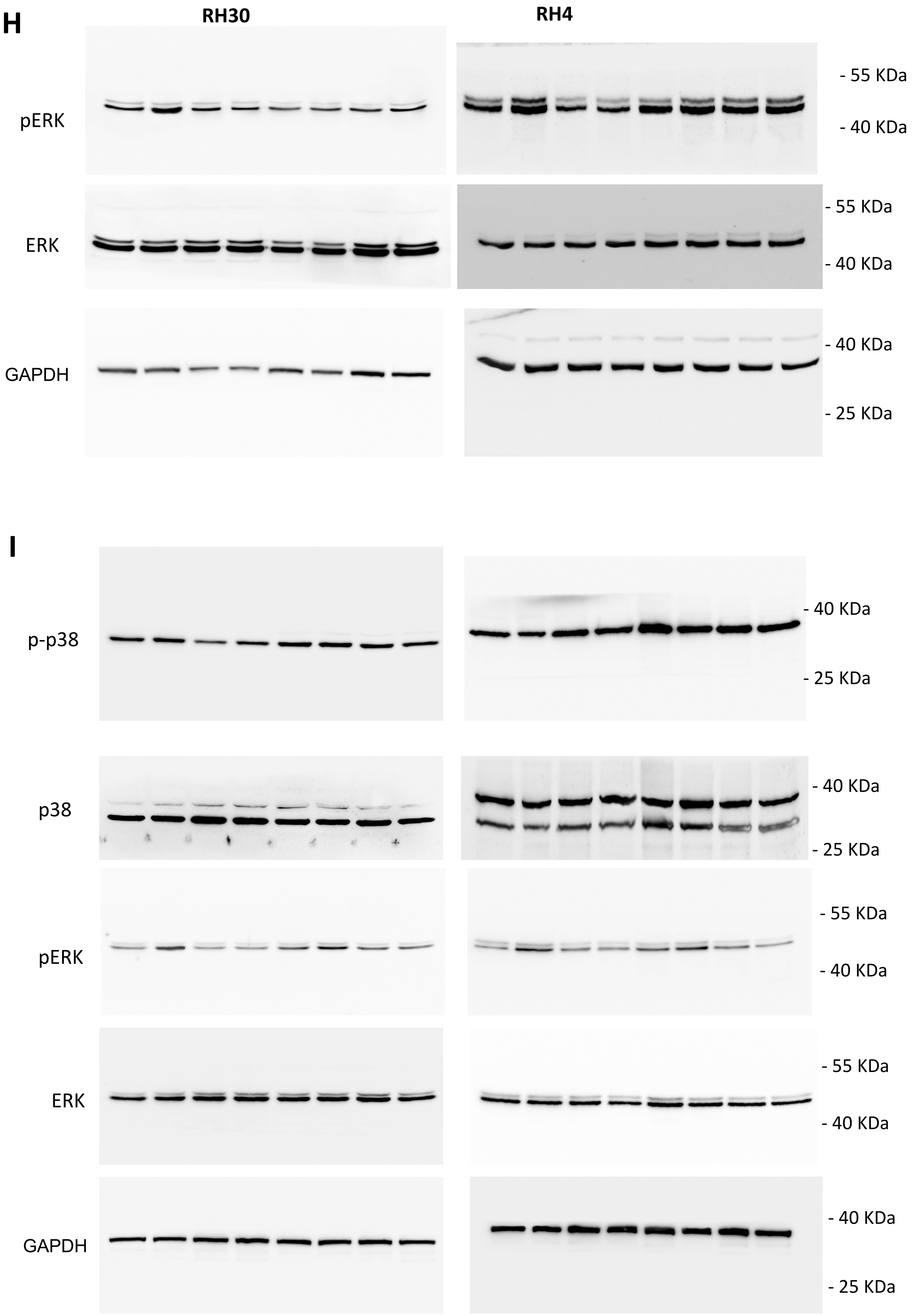

H
RH30
RH4
pERK
- 55 KDa
- 40 KDa
ERK
- 55 KDa
- 40 KDa
GAPDH
- 40 KDa
- 25 KDa
I
p-p38
- 40 KDa
- 25 KDa
p38
- 40 KDa
- 25 KDa
pERK
- 55 KDa
- 40 KDa
ERK
- 55 KDa
- 40 KDa
GAPDH
- 40 KDa
- 25 KDa

Figure S4

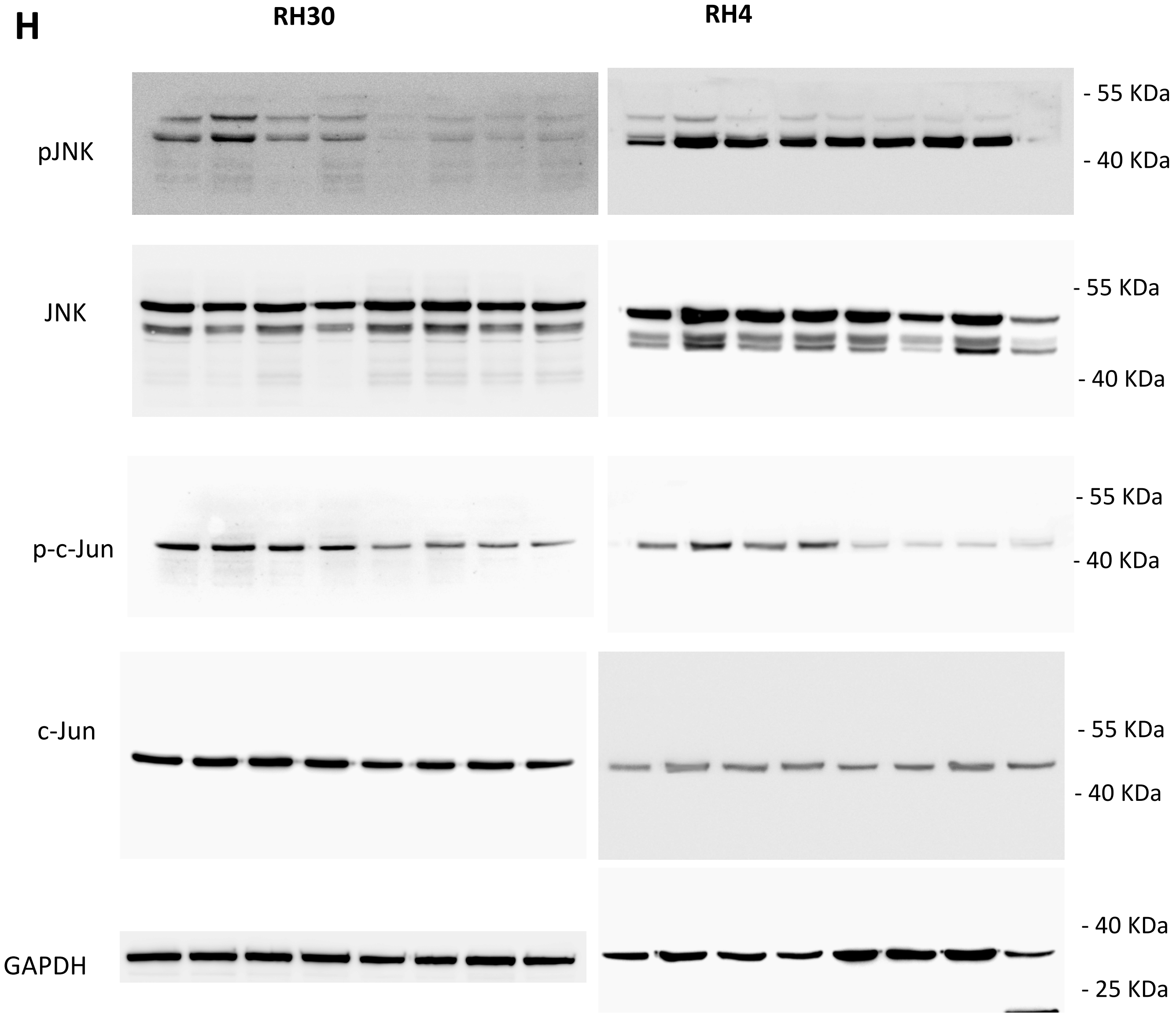
H
RH30
RH4
pJNK
JNK
p-c-Jun
c-Jun
GAPDH
- 55 KDa
- 40 KDa
- 55 KDa
- 40 KDa
- 55 KDa
- 40 KDa
- 55 KDa
- 40 KDa
- 40 KDa
- 25 KDa
